# Early metastasis is characterized by Gr1+ cell dysregulation and is inhibited by immunomodulatory nanoparticles

**DOI:** 10.1002/1878-0261.70040

**Published:** 2025-04-23

**Authors:** Jeffrey A. Ma, Sophia M. Orbach, Kate V. Griffin, Kathryn Kang, Yining Zhang, Rebecca S. Pereles, Ian A. Schrack, Guillermo Escalona, Jacqueline S. Jeruss, Lonnie D. Shea

**Affiliations:** ^1^ Department of Biomedical Engineering University of Michigan Ann Arbor MI USA; ^2^ Department of Chemical Engineering University of Michigan Ann Arbor MI USA; ^3^ Department of Surgery University of Michigan Ann Arbor MI USA; ^4^ Department of Pathology University of Michigan Ann Arbor MI USA

**Keywords:** Gr1+ myeloid cells, immunosuppression, metastatic niche, myeloid‐derived suppressor cells, nanoparticles

## Abstract

Cancer metastasis is supported by dysregulated myeloid‐derived suppressor cells, but myeloid cells are highly heterogeneous populations with distinct subsets that may support or inhibit tumor cell colonization. We hypothesize that Gr1+ myeloid cells transform in phenotype to support tumor cell colonization at the metastatic niche. In the 4T1 model of metastatic breast cancer, we investigate changes in the composition and phenotype of Gr1+ cells between premetastatic disease and early metastasis. Gr1+ cells in the lung were found to transition towards immunosuppressive and tumor‐supportive phenotypes with disease progression. While the composition of myeloid cells becomes dysregulated systemically, cells in the blood do not develop tumor‐supportive phenotypes, indicating that protumor functions are specific to the lung. *In vitro* assays demonstrate that Gr1+ cells from early metastatic lungs support tumor cell survival, migration, and proliferation, which is linked to chitinase‐3‐like protein 1 (CHI3L1) signaling. The intravenous injection of polymeric nanoparticles reprograms Gr1+ cell phenotypes, reduces the secretion of CHI3L1, and inhibits metastasis. These findings indicate that dysregulated Gr1+ cells are a therapeutic target for early metastasis and can be targeted with polymeric nanoparticles.

## Graphical abstract

Breast cancer metastasis is associated with myeloid cell dysregulation and the lung‐specific accumulation of tumor‐supportive Gr1+ cells. Gr1+ cells support metastasis, in part, through a CHI3L1‐mediated mechanism, which can be targeted and inhibited with cargo‐free, polymeric nanoparticles.

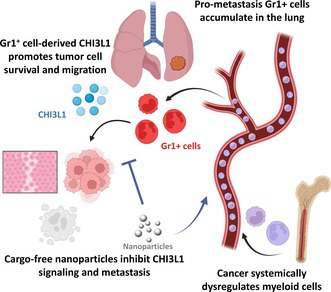

**Abbreviations**
CHI3L1: chitinase‐3‐like protein 1
NP: nanoparticle
PLG: poly(lactide‐co‐glycolide)
ROS: reactive oxygen species

## 1 Introduction

Breast cancer is the most common cancer among women in the United States [1]. While advances in the early detection of breast cancer have improved prognoses for localized tumors, cancers that have metastasized and spread to distant organs remain difficult to treat and are the primary cause of cancer‐associated mortality for solid tumors [2, 3]. The approval of T‐cell immunotherapies, such as checkpoint blockade inhibitors, has provided novel therapeutic options for metastatic cancers that have been able to induce complete remission in certain cases [4]. However, the cellular microenvironment at metastatic sites, known as the metastatic niche, confers resistance by shielding disseminated tumor cells from antitumor responses [5]. Myeloid cells in the metastatic niche can both directly and indirectly promote tumor cell accumulation by secreting cytokines that promote tumor cell recruitment or suppress T‐cell cytotoxicity [6]. Identifying specific populations and pathways through which myeloid cells support tumor cell survival may provide novel therapeutic strategies for disrupting the metastatic niche.

Myeloid immune cells play a significant role in the metastatic niche. Immature, dysregulated myeloid cells are released into circulation and accumulate at distal organs in response to signals released from the primary tumor [6, 7]. Dysregulated myeloid cells, which are often referred to as myeloid‐derived suppressor cells, can directly promote tumor cell colonization and the depletion of myeloid cells has been reported to inhibit metastasis in experimental settings [8, 9, 10]. Historically, myeloid‐derived suppressor cells have been identified with surface markers such as Gr1+, which encompasses both monocytic Ly6C+ populations and granulocytic Ly6G+ populations [11]. However, advances in transcriptomic technologies have revealed significant heterogeneity within Gr1+ cells. While Gr1+ cells associated with advanced metastases are dominated by subsets with tumor‐supportive phenotypes, Gr1+ cells also include mature neutrophil and monocyte populations that can induce tumor cell apoptosis and promote immune surveillance [12]. As a result, while myeloid cell dysregulation is associated with disease progression, the accumulation of myeloid cells does not necessarily indicate that tumor cells can engraft. Single‐cell RNA sequencing has shown that the balance of protumor and antitumor myeloid cell subsets shifts between the premetastatic niche, where tumor‐inhibiting populations dominate, and an early metastatic niche, where tumor‐supportive cells promote tumor cell engraftment [13, 14, 15]. This suggests that changes in myeloid cell composition and function are responsible for successful colonization and motivates further investigation into how myeloid cells interact with tumor cells to permit metastasis.

The presence of tumor‐supportive and tumor‐suppressive subsets in myeloid cells necessitates the identification of novel therapeutics to specifically target protumor phenotypes. While therapies broadly depleting or modulating myeloid cells have inhibited metastasis in experimental models, these techniques have largely failed to translate to the clinic [12, 16]. For example, inhibition of CCL2 was found to reduce myeloid‐derived suppressor cell accumulation in mice, but the nonspecific inhibition of immune signaling was found to result in toxicity and compromised immune function [17, 18]. Similarly, while depletion of myeloid‐derived suppressor cells alleviates immunosuppression in advanced metastases, the depletion of Gr1+ myeloid cells during premetastatic disease exacerbated metastasis, suggesting that this treatment depleted antitumor myeloid cell populations that were initially suppressing metastatic colonization [14, 19, 20]. As such, a need was identified for therapeutic strategies that aim to skew myeloid cells towards antitumor phenotypes without broadly depleting myeloid cells or disrupting physiological immune signaling. We have previously found that cargo‐free, poly(lactide‐co‐glycolide) (PLG) nanoparticles (NPs) modify immune cell trafficking and phenotype to stimulate adaptive immune responses to clear established metastatic lesions [21, 22]. We hypothesize that we can utilize nanoparticles to influence myeloid cell accumulation and phenotypes in the early stages of metastasis to directly inhibit tumor cell colonization.

In this report, we investigate the phenotypic evolution of Gr1+ myeloid cells in 4T1 tumor‐bearing mice between the premetastatic niche, distinguished by immune cell accumulation without detectable metastases, and the early metastatic niche, following the colonization of tumor cells. Changes in Gr1+ cell composition and phenotype in the lung and circulating blood are characterized via transcriptomic and surface marker analysis to compare tissue specific and systemic trends in Gr1+ cell phenotype. The influence of lung‐derived Gr1+ cells on tumor cell colonization is characterized *ex vivo* through tumor cell assays. We next characterize the secretome of Gr1+ cells and perform pathway analysis to identify specific pathways through which Gr1+ cells promote tumor cell colonization. Finally, we investigate the mechanisms by which PLG nanoparticles inhibit oncogenic signaling and maturation of the metastatic niche. Collectively, we identify a lung‐specific accumulation of tumor‐supportive myeloid cell subsets that can be reprogrammed via the administration of PLG nanoparticles and demonstrate the potential of nanoparticles as a myeloid cell‐targeting immunotherapy.

## 2 Materials and methods

### 2.1 Cell culture

4T1‐luc2‐tdTomato cells (RRID: CVCL_5J46) were cultured in RPMI 1640 Medium (Gibco, Carlsbad, CA, USA) supplemented with 10% fetal bovine serum (Avantor, Radnor, PA, USA) at 37 °C and 5% CO_2_ for 5 days prior to inoculation. 4T1 cells were originally purchased from Perkin Elmer (Waltham, MA, USA) and validated by short tandem repeat profiling performed by ATCC (Manassas, VA, USA). Briefly, 18 mouse short tandem repeat loci and two additional markers to screen for human or African green monkey species contamination were analyzed. Each sample was processed using the ABI Prism 3500xl Genetic Analyzer and analyzed using the genemapper id‐x v1.2 software (Applied Biosystems, Waltham, MA, USA). Cells were tested within 3 years of use and confirmed to have > 80% match with the ATCC reference cell line (CCRL‐2539‐LUC2). Cells for all experiments were confirmed to be mycoplasma‐free via testing performed by ATCC.

To prepare cells for inoculation, 4T1 cells were enzymatically detached from tissue culture flasks with 0.25% trypsin–EDTA (Gibco) and washed in culture media. Cells were centrifuged at 500 ***g*** for 5 min and resuspended in Dulbecco's phosphate‐buffered saline (DPBS; Gibco) at a density of 10 × 10^6^ cells·mL^−1^ for inoculation.

### 2.2 Animal studies and tumor model

Fifty microliter of cell suspension (10 × 10^6^ cells·mL^−1^) was surgically inoculated into the fourth right mammary fat pad of 8‐week‐old female BALB/c mice purchased from the Jackson Laboratory (Bar Harbor, ME, USA; strain #000651). Mice were housed in ventilated cages with corn cob bedding with 5 mice per cage in the vivarium managed by the University of Michigan Animal Care & Use Program. Animals were provided with Teklad 7912 chow and water *ad libitum*. Mice were handled under sterile conditions, using Spor‐Klenz to sterilize any gloves or surfaces in contact with animals. Mice were anesthetized with 2.5% v/v isoflurane, and the surgical site was prepared by sterilization with Betadine (Purdue Products, Stamford, CT, USA) and ethanol. Surgical scissors were used to create a 5 mm incision to expose the fat pad for injection, and following injection, the incision was closed with surgical wound clips (Roboz, Gaithersburg, MD, USA). 5 mg·kg^−1^ carprofen was administered immediately before and 24 h after surgery as an analgesic, and surgical sites were monitored for 7 days after surgery. For Gr1 depletion studies, mice were intraperitoneally injected with 100 μg of anti‐Gr1 antibody (Bio X Cell, Lebanon, NH, USA; clone RB6‐8C5) on days 1 and 6 after inoculation. For nanoparticle treatment studies, mice were intravenously injected 1 mg of nanoparticles on days 1, 4, 7, and 9 after inoculation. All animal studies were approved by the University of Michigan Institutional Animal Care and Use Committee under protocols #00009715 or #00011457.

### 2.3 Nanoparticle fabrication and injections

Nanoparticles were fabricated via a single‐emulsion method as previously described [23]. Briefly, 0.55–0.75 dL·g^−1^ ester‐terminated poly(lactide‐co‐glycolide) (Evonik, Birmingham, AL, USA) was dissolved in dichloromethane (Sigma, Burlington, MA, USA) at a concentration of 200 mg·mL^−1^ and added to 2% w/v poly(vinyl alcohol) in water at a 1 : 5 ratio. For experiments with Cy5.5‐labeled nanoparticles, poly(lactide‐co‐glycolide) was conjugated to Cy5.5 using EDC‐NHS chemistry, and the conjugated polymer was mixed with unconjugated polymer at a 1 : 20 w/w ratio. The mixture was then sonicated at 100% amplitude for 30 s with a Cole‐Parmer Ultrasonic processor (Model XPS130). The emulsion was added to a bath of 0.5% w/v poly(vinyl alcohol) in water and allowed to evaporate overnight to remove dichloromethane. Nanoparticles were collected by centrifugation at 5000 ***g*** for 15 min and washed in 0.1 m sodium bicarbonate (Polysciences, Warrington, PA, USA) or Milli‐Q water for a total of five washes. Nanoparticles were resuspended at a concentration of approximately 20 mg·mL^−1^ in water supplemented with 4% w/v sucrose and 3% w/v mannitol (Sigma) as a cryoprotectant. The resulting suspension was frozen and lyophilized for 3 days before use. The size and zeta potential of the nanoparticles were characterized via dynamic light scattering using a Malvern Zetasizer Nano ZSP (Westborough, MA, USA).

To prepare nanoparticles for injection, lyophilized nanoparticles were resuspended in distilled water and centrifuged at 5000 ***g*** for 5 min to remove cryoprotectant. Nanoparticles were then resuspended in sterile DPBS at a concentration of 10 mg·mL^−1^ and passed through a 40 μm cell strainer to remove particulates. One hundred microliter of nanoparticle solution was intravenously injected into mice via 30 G needles (BD, Franklin Lakes, NJ, USA).

### 2.4 Tissue processing and cell isolation

Lungs were extracted from tumor‐free mice or tumor‐bearing mice 3 or 10 days after tumor inoculation. Lungs were mechanically and enzymatically digested using liberase TM (Roche, Pleasanton, CA, USA) under sterile conditions and filtered through a 70 μm cell strainer. Samples were then treated with ammonium‐chloride‐potassium lysing buffer (Gibco) for 5 min to lyse erythrocytes and washed with DPBS supplemented with 2 mm EDTA and 0.5% bovine serum albumin (cell isolation buffer). Blood samples were extracted via cardiac puncture and treated with lysing buffer to isolate leukocytes. To select for Gr1+ cells, the resulting single‐cell suspensions were processed with the mouse Myeloid‐Derived Suppressor Cell Isolation Kit (Miltenyi Biotec, Waltham, MA, USA) with minor modifications to the manufacturer protocol. Samples were stained with the anti‐Gr1 antibody and antibiotin microbeads then passed through an LS column. The resulting purified Gr1+ cells were then directly cocultured with 4T1 cells or splenic T cells that were isolated in a similar fashion using the mouse Pan T Cell Isolation Kit II (Miltenyi Biotec) following manufacturer instructions. Alternatively, Gr1+ cells were cultured in phenol‐red free, serum‐free RPMI 1640 (Gibco) supplemented with 0.5 mg·mL^−1^ penicillin/streptomycin for 16 h to generate conditioned media. Conditioned media was then filtered to remove cells and debris and frozen at −80 °C once before use in assays.

### 2.5 Flow cytometry

Single‐cell suspensions were prepared from lungs and blood as described in Section 2.4. For ROS staining, single‐cell suspensions were simultaneously stimulated with the Biolegend Cell Activation Cocktail without Brefeldin A and stained with dihydrorhodamine 123 for 20 min (Thermofisher, Waltham, MA, USA). Single‐cell suspensions were then washed and resuspended in DPBS and stained with the Zombie Violet Fixable Viability Kit (Biolegend, San Diego, CA, USA) following manufacturer instructions. Cells were then washed with cell isolation buffer and stained with anti‐CD16/32 (clone 93) to prevent nonspecific antibody binding. Samples were stained with the following antibodies: BV510‐CD11b (clone M1/70), BV711‐Ly6G (clone 1A8), FITC‐Ly6C (clone HK‐1.4), PE‐CD43 (clone 1B11), AF700‐CD45 (clone 30‐F11), BV510‐CD4 (clone RM4‐5), AF594‐CD8a (clone 53–6.7), and AF700‐CD3 (clone 17A2). For intracellular staining, samples were fixed and permeabilized using the Biolegend True‐Nuclear Transcription Factor Buffer set. Samples were then stained with BV711‐GATA3 (BD, clone L50‐832), AF488‐RORγt (Thermofisher, clone B2D), PE‐Tbet (clone 4B10), and AF647‐FoxP3 (clone MF‐14). All antibodies were purchased from Biolegend unless specified otherwise. Samples were then washed with cell isolation buffer and filtered using a 40 μm cell strainer prior to analysis. For experiments analyzing the absolute number of cells per tissue, CountBright Plus Absolute Counting Beads (Thermofisher) were added to each sample immediately before analysis following manufacturer instructions. Samples were analyzed with a Bio‐Rad ZE5 Cell Analyzer (Hercules, CA, USA). Data analysis was performed using flowjo software (BD).

### 2.6 RNA sequencing

Gr1+ cells were isolated from lungs at 3 and 10 days after inoculation as described in Section 2.4. Gr1+ cells were pelleted, frozen, and processed with the RNeasy Plus Mini spin column kit (Qiagen, Germantown, MD, USA) to isolate mRNA. Purified RNA was quantified using a Nanodrop 2000c (Thermofisher) and submitted to the University of Michigan Advanced Genomics Core for library preparation and sequencing. Samples were prepared following the QuantSeq 3′ mRNA‐seq protocol and sequenced on the Illumina NextSeq 550 sequencer. Raw sequencing counts were normalized, and differential gene expression was calculated using the deseq2 package [24]. Gene set enrichment analysis was performed using the gsea software (Broad Institute) to identify significantly upregulated pathways based on differentially expressed genes [25].

For single‐cell RNA sequencing experiments, single cells were isolated from NP‐treated and untreated lungs 14 days following inoculation as described in Section 2.4; cells from NP‐treated lung were sorted into NP+ and NP− populations using Cy5.5 fluorescent NPs. Sorted samples were fixed with 10× Chromium Next GEM Single Cell Fixed RNA Sample Preparation Kit per the manufacturers protocol. Fixed cells were sequenced by the University of Michigan Advanced Genomics Core with the 10× Flex Platform on the NovaSeq X 10B flow cell (300 cycle) at approximately 25 k reads per sample. Unfiltered outputs from CellRanger were processed per sample with SoupX, ddqcR, and DoubletFinder [26, 27, 28]. Data were merged, normalized with SCTransform, and integrated using the seurat v5 pipeline [29, 30]. Dimensional reduction via principal component analysis and uniform manifold approximation and projection clustered the data into cell types; neutrophils were identified using canonical marker genes and subset to compare NP+, NP−, and untreated neutrophils. Differentially expressed genes were calculated using Seurat with minimum percent expression and log_2_FC threshold of 0.25. Differentially expressed genes with adjusted *P*‐value < 0.05 were considered significant, with positive log_2_FC considered upregulated and negative considered downregulated. Up‐ and downregulated genes were input into hypeR for hypergeometric testing with pathways from the Hallmark collection from MSigDB [25, 31, 32].

### 2.7 Tumor cell assays

Tumor cells were cocultured with isolated Gr1+ cells or cultured in Gr1+ cell‐conditioned media to assay tumor cell proliferation, apoptosis, and migration. For studies on the effects of CHI3L1, conditioned media was supplemented with 1 μg·mL^−1^ of recombinant CHI3L1 (R&D Systems, Minneapolis, MN, USA) or 10 μg·mL^−1^ of anti‐CHI3L1 (Sigma). To measure tumor cell apoptosis, 1 × 10^4^ 4T1 cells were seeded in a flat‐bottom 96‐well plate and allowed to adhere overnight. 4T1 cells were then cultured with Gr1+ cells (1 : 5 ratio) or Gr1+ cell‐conditioned media for 16 h. Wells were washed with DPBS to remove nonadherent Gr1+ cells and stained with annexin V‐FITC to identify apoptotic cells (Thermofisher). Wells were imaged using a Zeiss Axio Observer Z1 (Danvers, MA, USA) with a 10× objective. Three random, nonoverlapping images were taken in each well and quantified using fiji software [33].

To measure tumor cell migration, 3.75 × 10^5^ 4T1 cells were plated in a 24‐well plate and allowed to grow to confluence. A scratch was made down the middle of each well using a 200 μL pipette tip, and the wells were washed twice with DPBS. Prior to adding Gr1+ cells or conditioned media, three locations along the scratch were imaged using the Zeiss Axio Observer Z1. Tumor cells were cultured with Gr1+ cells (1 : 5 ratio) or Gr1+ cell‐conditioned media for 5 h. Wells were washed with DPBS twice to remove nonadherent cells, and the same locations were re‐imaged. The relative closure at each imaged location was quantified using fiji software.

To measure tumor cell proliferation, 1 × 10^4^ 4T1 cells were seeded in a black, clear‐bottom 96‐well plate and allowed to adhere overnight. tdTomato fluorescence (554/581 excitation/emission), quantifying the number of 4T1 cells in each well, was measured using a Biotek Synergy H1 Microplate Reader (Lexington, MA, USA) at 0, 24, and 48 h after the addition of Gr1+ cells (1 : 5 ratio) or conditioned media.

### 2.8 T‐cell assays

Splenic T cells were isolated from tumor‐free mice as described in Section 2.4 and stained with CellTrace Far Red (Thermofisher) for 10 min in DPBS and quenched with RPMI 1640 media supplemented with serum. Stained T cells were plated in a flat‐bottom, low‐attachment 96‐well plate at a density of 1 × 10^5^ cells/well. Gr1+ cells (1 : 1 ratio) and 2 μL of Dynabeads were added to each well, and cells were cultured at 37 °C and 5% CO_2_ for 72 h. Cells were removed from wells, washed to remove Dynabeads, and prepared for flow cytometry as described above.

### 2.9 Protein array and ELISA

Analysis of protein and CHI3L1 content in Gr1+ cell‐conditioned media was performed using the Proteome Profiler Mouse XL Cytokine Array and the Mouse Chitinase 3‐like 1/YKL‐40 Quantikine ELISA Kit from R&D Systems, respectively. Assays were performed on undiluted Gr1+ cell‐conditioned media following manufacturer instructions. The protein array membrane was imaged using the ChemiDoc Imaging System (Bio‐Rad, Hercules, CA, USA) and analyzed using fiji. The ELISA plate was measured using a Biotek Synergy H1 Microplate Reader at 450/540 excitation/emission.

### 2.10 Chemotaxis assay

Gr1+ cells were isolated from the spleens of tumor‐free mice and isolated using a Gr1+ isolation kit as described in Section 2.4. Gr1+ cells were plated apically to conditioned media generated from unsorted lung cells 3 and 10 days after inoculation. Gr1+ cells were plated in RPMI 1640 supplemented with glucose, sodium bicarbonate, sodium pyruvate, 4‐(2‐hydroxyethyl)‐1‐piperazineethanesulfonic acid, and l‐glutamine (ATCC modifications) to preserve Gr1+ cell viability. Gr1+ cells were allowed to migrate for 2 h through a transwell with 3.0 μm pores (Corning, Corning, NY, USA). Following migration, cells were quantified by flow cytometry as described above with the addition of CountBright Plus Absolute Counting Beads (Invitrogen, Waltham, MA, USA) to quantify the number of migrated cells.

### 2.11 Bioluminescence imaging of tissue

Lung metastases were imaged and quantified using an IVIS Lumina LTE imaging system (Caliper Life Sciences, Hopkinton, MA, USA). Following explant, lungs were incubated in serum‐free RPMI 1640 containing 630 μm d‐luciferin (Perkin Elmer) for 8 min and imaged with the IVIS using automatic exposure settings. Images taken by the IVIS were quantified using the living image software from Caliper Life Sciences.

### 2.12 Transcription factor activity assays

Transcription factor activity in tumor cells was measured using the TRACER technique [34, 35]. Briefly, 4T1 cells were transfected with 10 MOI of lentivirus for transcription factor activity reporters. Reporters contain a transcription factor response element upstream of a luciferase promoter, such that increased transcription factor activity increases luciferase expression and thus luminescence. After the addition of lentivirus, 4T1 cells were cultured for 2 days to allow lentiviral gene expression. Cells were then cultured in Gr1+ cell‐conditioned media, and luciferase activity was measured using an IVIS for 2 days. Luminescence was quantified using the living image Software and normalized to a minimal promoter.

### 2.13 Statistics

Statistical analyses were performed using graphpad prism 10 software (Boston, MA, USA) with unpaired Student's *t*‐tests. Transcriptomic analyses were performed using R and the significance of GSEA pathway scores were determined with Student's *t*‐tests using the Bonferroni multiple hypothesis correction with α = 0.01. *P*‐values < 0.05 were considered statistically significant. Error bars on all plots indicate mean ± standard deviation.

## 3 Results

### 3.1 Myeloid cells in the metastatic niche shift towards granulocytic, immunosuppressive phenotypes

**Fig. 1:**
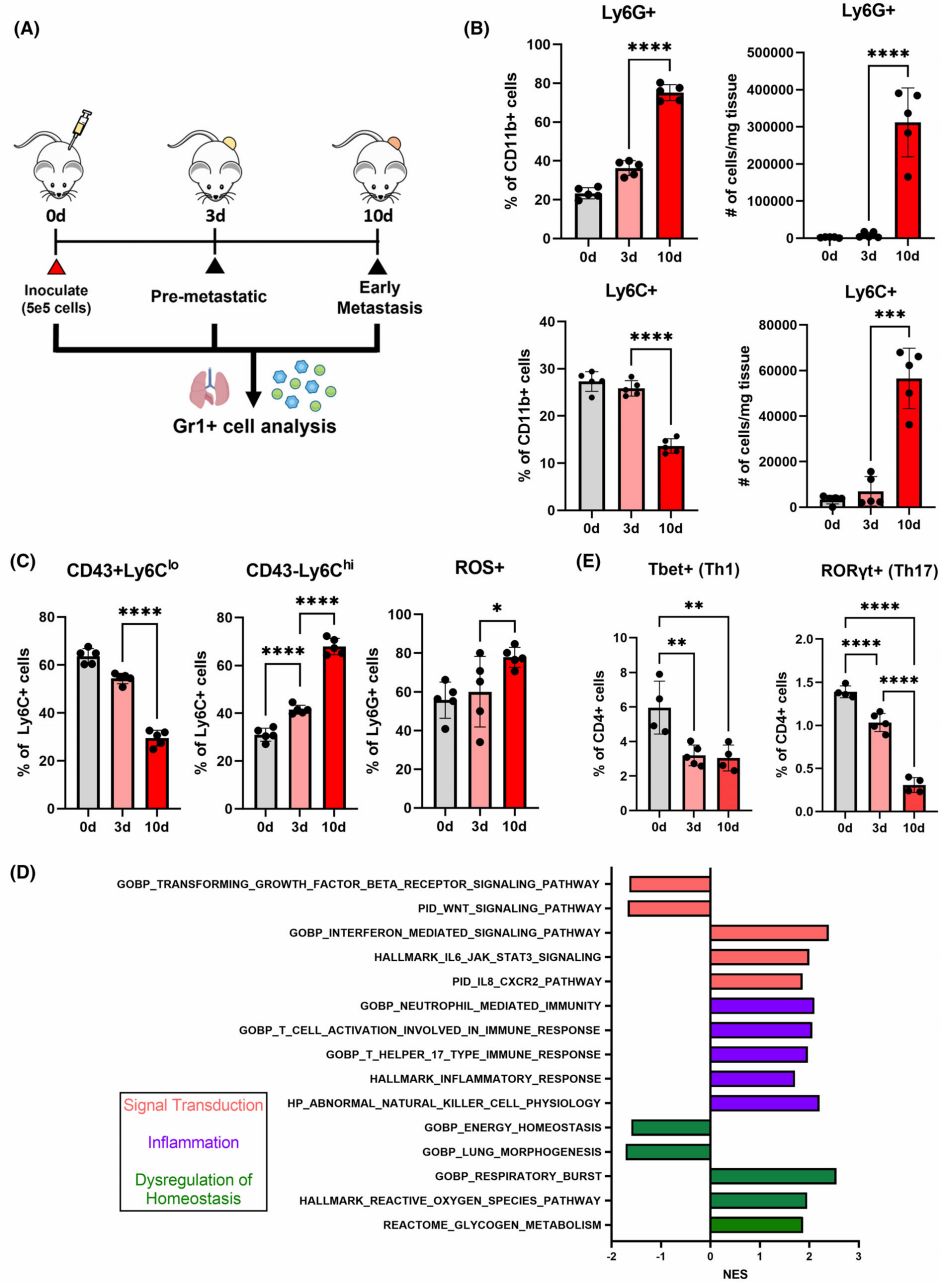
Gr1+ cells change in composition and phenotype in the early stages of metastatic disease. (A) Schematic of Gr1+ cell analysis in tumor‐free (0 days—0d), premetastatic (3 days—3d), and early metastatic (10 days—10d) lungs. (B) Myeloid cells at the lung are predominantly Ly6C+ monocytes in healthy and premetastatic lungs but are supplanted by Ly6G+ neutrophils in early metastatic lungs. (C) Gr1+ cell subsets undergo tumor‐supportive shifts. The proportion of CD43+Ly6Clo classical monocytes decrease, and the proportions of CD43‐Ly6Chi classical monocytes and reactive oxygen species (ROS)‐expressing immunosuppressive neutrophils increase with disease progression. (D) Bulk RNA sequencing demonstrates that neutrophil and inflammation‐associated gene pathways are upregulated in Gr1+ cells from metastatic lungs relative to premetastatic Gr1+ cells (*n* = 3 biological replicates per condition). (E) CD4 T cells with Th1 (Tbet+) or Th17 (RORγt+) phenotypes decrease in early metastatic lungs compared to healthy or premetastatic lungs. Two‐tailed unpaired *t*‐tests assuming unequal variance were performed for single comparisons between two conditions, *P ≤ 0.05, **P ≤ 0.01, ****P ≤ 0.0001. Bars indicate mean ± standard deviation with *n* = 5 biological replicates unless otherwise noted.

We hypothesize that the development of the metastatic niche is characterized by the accumulation of tumor‐supportive myeloid cells and thus investigated myeloid cell dynamics at the lung in the 4T1 model of metastatic breast cancer. We analyzed the immune composition of the lung at 3 days after inoculation, which corresponds to a premetastatic niche, and at 10 days after inoculation, when the lung has developed into an early‐stage metastatic niche with tumor cell seeding (Fig. 1A) [14]. Gr1+ myeloid cells in 3 days lungs demonstrated early signs of immune dysregulation but were not significantly different from tumor‐free lungs (Fig. 1B, Figs S1 and S2A). At this stage, the CD11b+ myeloid compartment was predominantly composed of Ly6C+ monocytes (25.8%) and Ly6G+ neutrophils (36.3%). From 3 to 10 days, there is a significant 2.07‐fold increase in the proportion of neutrophils, which constitute 75.2% of CD11b + myeloid cells at 10 days. There is a corresponding 1.89‐fold decrease in the proportion of Ly6C+ monocytes, which constituted only 13.6% of CD11b+ cells at 10 days. Notably, this observed change in proportion is driven by the overwhelming accumulation of neutrophils (Fig. 1B). While the absolute number of neutrophils and monocytes per mg of lung both increase with disease progression, the influx of neutrophils is substantially greater than that of monocytes. The number of neutrophils per mg of lung increases 35.9‐fold between 3 and 10 days, while the number of monocytes increases 8.13‐fold. Neutrophils and monocytes also undergo phenotypic changes between the 3 and 10 days (Fig. 1C, Fig. S2B). In 3 days lungs, Ly6C+ cells skew towards CD43+Ly6C^lo^ nonclassical monocytes (54.4%), which play roles in immune surveillance [36]. At this stage, CD43‐Ly6C^hi^ classical monocytes, which can differentiate into tumor‐associated macrophages, constitute 41.5% of Ly6C+ monocytes. Ten days lungs are instead dominated by classical monocytes (67.9%, 1.63‐fold increase from 3 days), which supplant nonclassical monocytes (29.5%, 1.85‐fold decrease from 3 days). While the number of nonclassical monocytes per mg lung increases at 10 days relative to 3 days (2.91‐fold increase), this increase is overshadowed by the increase in the number of classical monocytes (17.0‐fold, Fig. S3A). Similarly, the proportion of neutrophils expressing reactive oxygen species (ROS) increases with disease progression, which is reported to be associated with metastasis and immunosuppression [37, 38, 39]. At 10 days, a higher proportion of Ly6G+ neutrophils express ROS (77.9%) compared to neutrophils in 3 days lungs (60.1%, 1.30‐fold increase).

We next characterized transcriptomic differences in Gr1+ cells to investigate phenotypes associated with shifts in surface marker expression. Gr1+ cells were isolated from 3 and 10 days lungs and analyzed by bulk RNA sequencing. Genes associated with granulocytic inflammation (Ngp, Ltf, Stfa3, Camp) were significantly upregulated in 10 days Gr1+ cells compared to 3 days (Fig. S3B), consistent with the observed increase in Ly6G+ neutrophils (Fig. 1B) [40]. We performed gene set enrichment analysis and found that pathways associated with neutrophil inflammation and recruitment (GOBP Neutrophil Mediated Immunity, PID IL8 CXCR2 Pathway) were highly upregulated in Gr1+ cells at 10 days (Fig. 1D). Interestingly, 10 days Gr1+ cells upregulated pathways associated with both T‐cell activation (GOBP T‐Cell Activation Involved in Immune Response and GOBP T Helper 17 Type Immune Response) and ROS‐mediated suppression of T cells (Hallmark Reactive Oxygen Species Pathway). While dysregulated Gr1+ cells are known to drive T‐cell suppression, healthy Gr1+ cells promote immune surveillance, suggesting that Gr1+ cells may be transitioning from immune‐stimulating to immunosuppressive phenotypes at the early metastatic stage [7]. To determine whether the early metastatic niche is immunosuppressive overall, we investigated *in vivo* T‐cell phenotypes in the lung and found that the proportion of Th1 (T‐bet+) and Th17 (RORγt+) in the lung at 10 days decreased relative to 0 or 3 days lungs (1.96‐fold and 3.45‐fold decreases, respectively; Fig. 1E, Fig. S2C). The reduction in antitumor T‐cell phenotypes suggests that the metastatic niche is more suppressive at 10 days compared to the lung microenvironment at earlier stages of disease progression [41]. Collectively, these data indicate that Gr1+ cells in the early metastatic niche transition in composition and phenotype with disease progression and shift towards subsets that are associated with tumor‐supportive and immunosuppressive phenotypes.

### 3.2 Gr1+ cell accumulation at the lung is distinct from systemic immune dysregulation

**Fig. 2:**
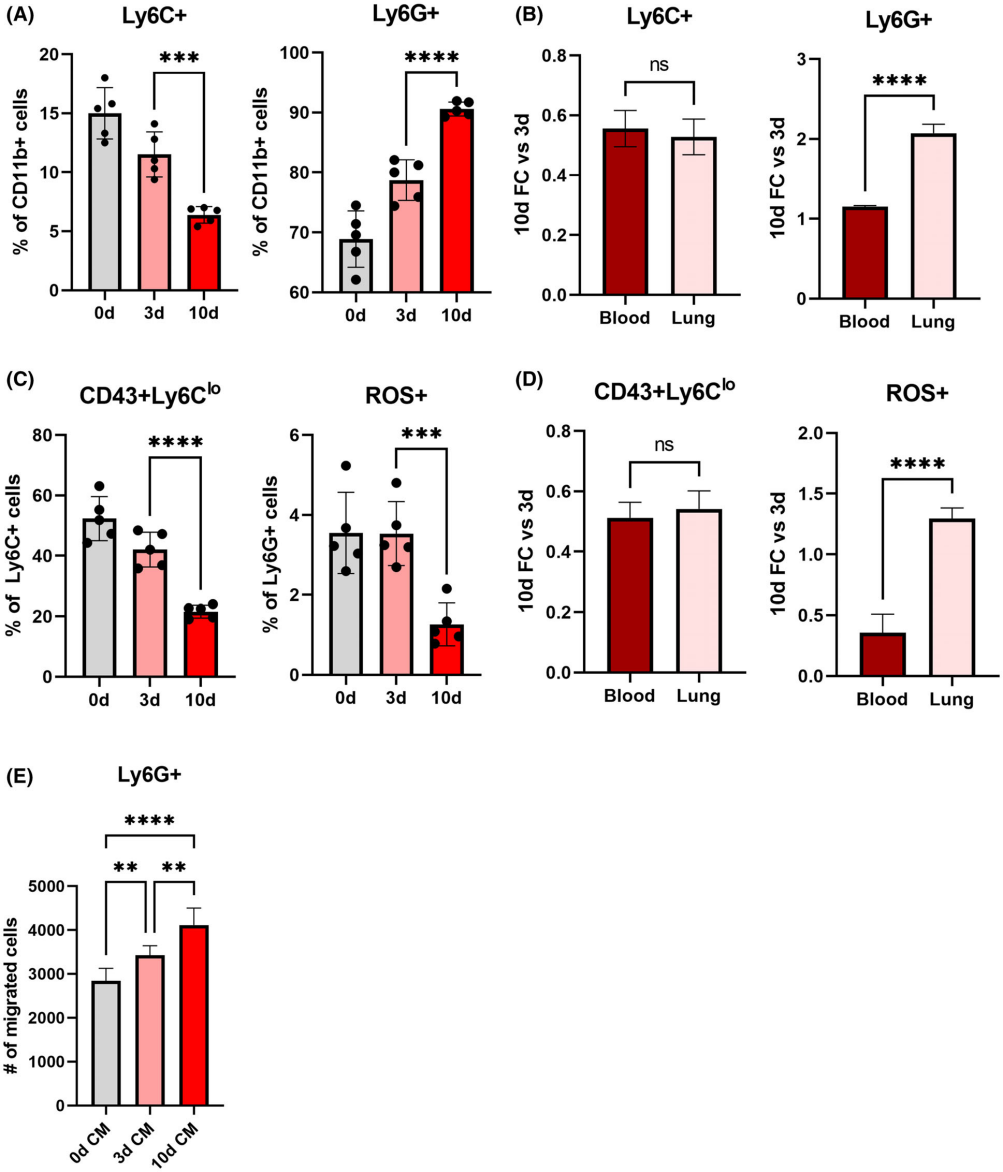
The preferential recruitment of neutrophils and changes in circulating myeloid populations drives immune dysregulation at the lung. (A) In circulating blood, the proportion of Ly6C+ monocytes decreases and the proportion of Ly6G+ neutrophils increases with disease progression from 0 days (0d) to 10 days after inoculation (10d). (B) The fold change of Ly6C+ monocytes in the lung from premetastatic to early metastatic disease is identical to the change in blood, but the fold change increase of Ly6G+ neutrophils in the lung is significantly higher than what is observed in circulation. (C) The proportion of nonclassical monocytes (CD43+) and reactive oxygen species (ROS)‐expressing neutrophils decreases in the blood with disease progression. (D) While there is no enrichment of nonclassical monocytes in the lung compared to the blood, the proportion of ROS‐expressing neutrophils increases in the lung while decreasing in the blood from 3 to 10 days. (E) An increased number of Ly6G+ neutrophils migrate in response to conditioned media (CM) generated from early metastatic lungs, compared to premetastatic or healthy lungs. Two‐tailed unpaired *t*‐tests assuming unequal variance were performed for single comparisons between two conditions, **P ≤ 0.01, ***P ≤ 0.001, ****P ≤ 0.0001. Bars indicate mean ± standard deviation with *n* = 5 biological replicates for all figures.

Immune dysregulation by tumors systemically alters immune populations and contributes to the establishment of the metastatic niche, but tumor cells preferentially metastasize to specific organs such as the lung [42, 43]. We hypothesize that metastasis is driven by tissue‐specific enrichment of tumor‐supportive myeloid cells. To compare systemic immune dysregulation to immune dysregulation specific to the lung, we characterized Gr1+ cells in the blood at 3 and 10 days after inoculation, corresponding to the premetastatic and early metastatic niche at the lung, respectively. Changes in the proportion of Ly6C+ monocytes and Ly6G+ neutrophils in the blood trended in the same direction as these populations in the lung, but not with the same magnitude (Fig. 2A, Fig. S4A). Between 3 and 10 days after inoculation, the proportion of monocytes in the blood decreased 1.25‐fold, and the proportion of neutrophils increased 1.15‐fold. The fold decrease in the proportion of monocytes in the lung from 3 to 10 days was identical to the fold decrease observed in the blood, indicating that changes in monocyte accumulation at the lung reflect systemic immune dysregulation (Fig. 2B). Conversely, the fold change increase of neutrophils in the blood did not account for the increase observed at the lung. Compared to the 1.15‐fold increase in neutrophils in the blood, there was a 2.07‐fold increase observed in the lung from 3 to 10 days, indicating that there may be preferential enrichment of neutrophils at the lung. Similarly, while changes in monocyte subsets in the blood reflected trends observed in the lung, changes in neutrophil phenotype observed in the lung were not observed in the blood. The proportion of Ly6G+ neutrophils expressing ROS decreased in the blood from 3 to 10 days (2.80‐fold decrease), but there was conversely a 1.30‐fold increase in the lung (Fig. 2C,D, Fig. S4B). This suggests that there are distinct factors driving neutrophil recruitment and phenotype at the lung beyond systemic immune dysregulation. To investigate the contribution of preferential recruitment, we performed a chemotaxis assay with conditioned media generated from 3 and 10 days lungs and found that chemokines secreted by metastatic lungs significantly increased Ly6G+ neutrophil migration (1.20‐fold increase; Fig. 2E). Overall, these data demonstrate that Gr1+ cells undergo a lung‐specific shift towards tumor‐supportive phenotypes with the maturation of the metastatic niche.

### 3.3 Lung Gr1+ cells promote tumor cell colonization

**Fig. 3:**
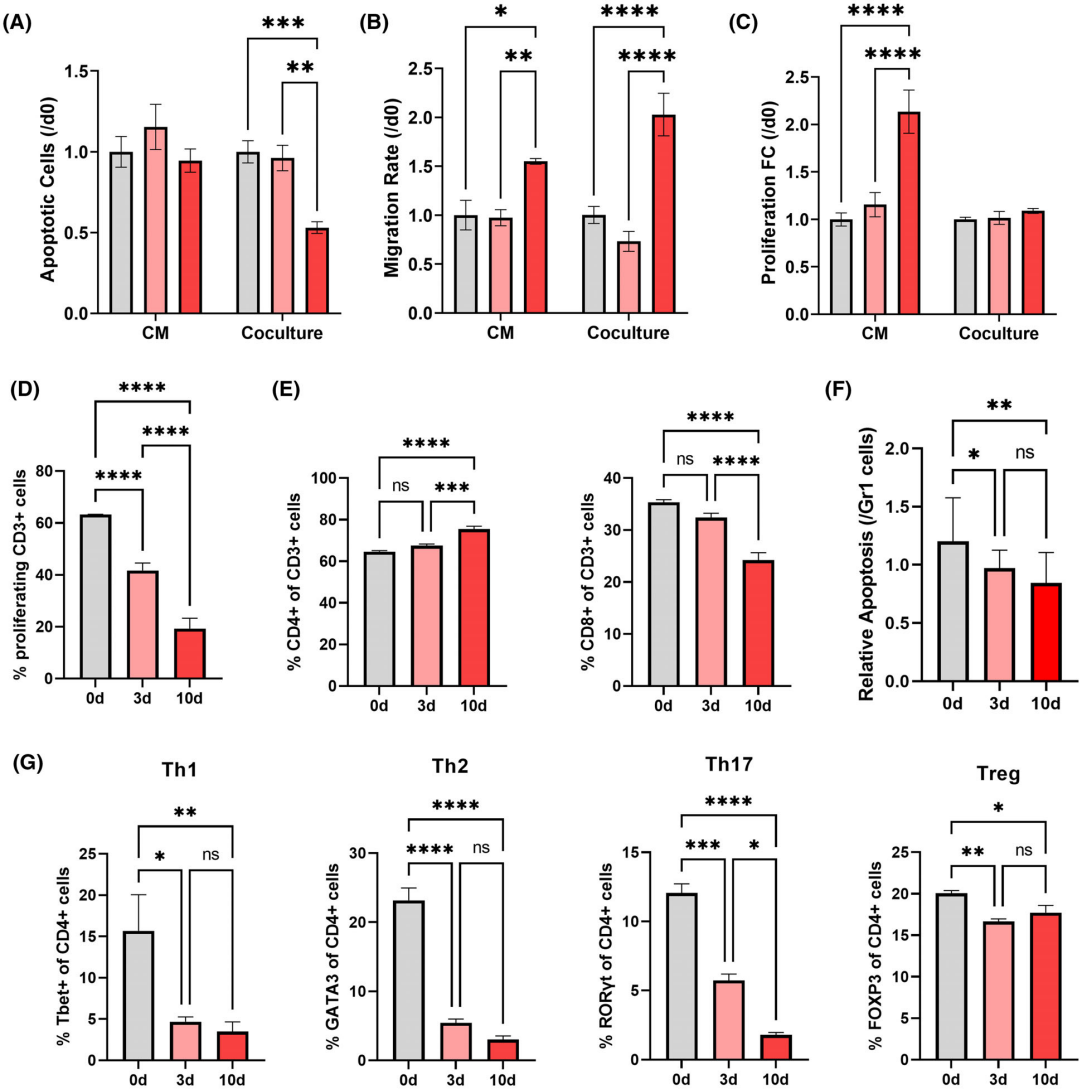
Gr1+ cells from early metastatic lungs support tumor cell colonization and suppress T‐cell responses. Early metastatic Gr1+ cell‐conditioned media (CM) or coculture with tumor cells from 0 days (0d), 3 days (3d), or 10 days after inoculation (10d) increases tumor cell (A) apoptosis, (B) motility, and (C) proliferation relative to premetastatic and healthy Gr1+ cells. (D) Gr1+ cells become more suppressive of T‐cell proliferation with disease progression. (E) T cells proliferating in coculture with Gr1+ cells tend to skew towards CD4+ cells over CD8a+ cells with disease progression. (F) T cells cocultured with Gr1+ cells from premetastatic or metastatic lungs are less cytotoxic than T cells cocultured with healthy Gr1+ cells and exhibit reduced induction of tumor cell apoptosis *in vitro*. (G) Gr1+ cells suppress expression of transcription factors associated with Th1, Th2, and Th17 CD4+ subsets. Two‐tailed unpaired *t*‐tests assuming unequal variance were performed for single comparisons between two conditions, *P ≤ 0.05, **P ≤ 0.01, ***P ≤ 0.001, ****P ≤ 0.0001; ns, no significance. Bars indicate mean ± standard deviation with at least *n* = 5 technical replicates for each assay with cells pooled from *n* = 5 biological replicates per condition.

We next investigated how the lung‐specific accumulation of granulocytic Gr1+ cells functionally influenced tumor cell colonization. We hypothesize that myeloid cells associated with the early metastatic niche at 10 days support metastasis both through direct effects on tumor cells as well as indirect effects on T‐cell immunity. To investigate the direct effects of Gr1+ cells, we isolated Gr1+ cells from the lung at 0, 3, and 10 days after inoculation for coculture with 4T1 cells or for generation of conditioned media, in which 4T1 cells were subsequently cultured. Ten days Gr1+ cells broadly supported tumor cell colonization and increased tumor cell migration, proliferation, and survival relative to 0 or 3 days Gr1+ cells (Fig. 3A–C). Conditioned media from 10 days Gr1+ cells increased tumor cell migration and proliferation, with a 1.59‐fold and 1.85‐fold increase over 3 days Gr1+ cells, respectively. While conditioned media did not influence tumor cell apoptosis, coculture with 10 days Gr1+ cells induced less tumor cell apoptosis (1.81‐fold decrease) than coculture with 3 days Gr1+ cells, indicating that Gr1+ cells may induce tumor cell apoptosis through cell–cell interactions. In coculture, 10 days Gr1+ cells also increased tumor cell migration (2.77‐fold increase) relative to 3 days Gr1+ cells. Unexpectedly, 10 days Gr1+ cells did not increase tumor cell proliferation. The lack of changes in tumor cell proliferation in coculture with Gr1+ cells may be due to cell–cell interactions, such as Gr1+ cell‐induced apoptosis or contact inhibition of growth. Three days Gr1+ cells did not significantly differ from 0 day Gr1+ cells in any assay, suggesting that premetastatic Gr1+ cells maintain antitumor phenotypes. Collectively, these results indicate that Gr1+ cells acquire tumor‐promoting phenotypes that are mediated, in part, through secreted factors.

Gr1+ cells also influence tumor cell fate indirectly by promoting or suppressing T‐cell surveillance. We cocultured T cells with Gr1+ cells to study the influence of premetastatic or early metastatic Gr1+ cells on T‐cell function. Gr1+ cells were progressively more suppressive of T‐cell proliferation with disease progression. T cells cocultured with 10 days Gr1+ cells exhibited a 2.16‐fold decrease in proliferation relative to 3 days Gr1+ cells (Fig. 3D). Gr1+ cell‐conditioned media did not influence T‐cell proliferation (Fig. S5). Coculture with 10 days Gr1+ cells skewed T cells towards CD4+ T cells (Fig. 3E). Among CD3+ T cells cultured with 10 days Gr1+ cells, there was a 1.17‐fold increase in the proportion of CD4+ T cells and a 1.46‐fold reduction in the proportion of CD8+ T cells relative to T cells cultured with 0 day Gr1+ cells. Accordingly, T cells cocultured with 10 days Gr1+ cells exhibited reduced cytotoxicity against 4T1 cells compared to T cells cultured with 0 day Gr1+ cells (1.43‐fold reduction) (Fig. 3F). In the CD4+ fraction, 10 days Gr1+ cells suppressed antitumor subsets and reduced the proportion of T cells expressing T‐bet (Th1), GATA3 (Th2), and RORγt (Th17) relative to T cells cocultured with 0 day Gr1+ cells (4.49‐fold, 7.67‐fold, and 6.74‐fold reductions, respectively), (Fig. 3G). Ten days Gr1+ cells modestly reduced the expression of FoxP3 in CD4+ T cells (1.13‐fold relative to 0 day Gr1+ cells). Taken together, this data indicates that Gr1+ cells acquire immunosuppressive phenotypes that distinguish the early metastatic niche.

### 3.4 Gr1+ cell‐derived CHI3L1 drives oncogenic phenotypes

**Fig. 4:**
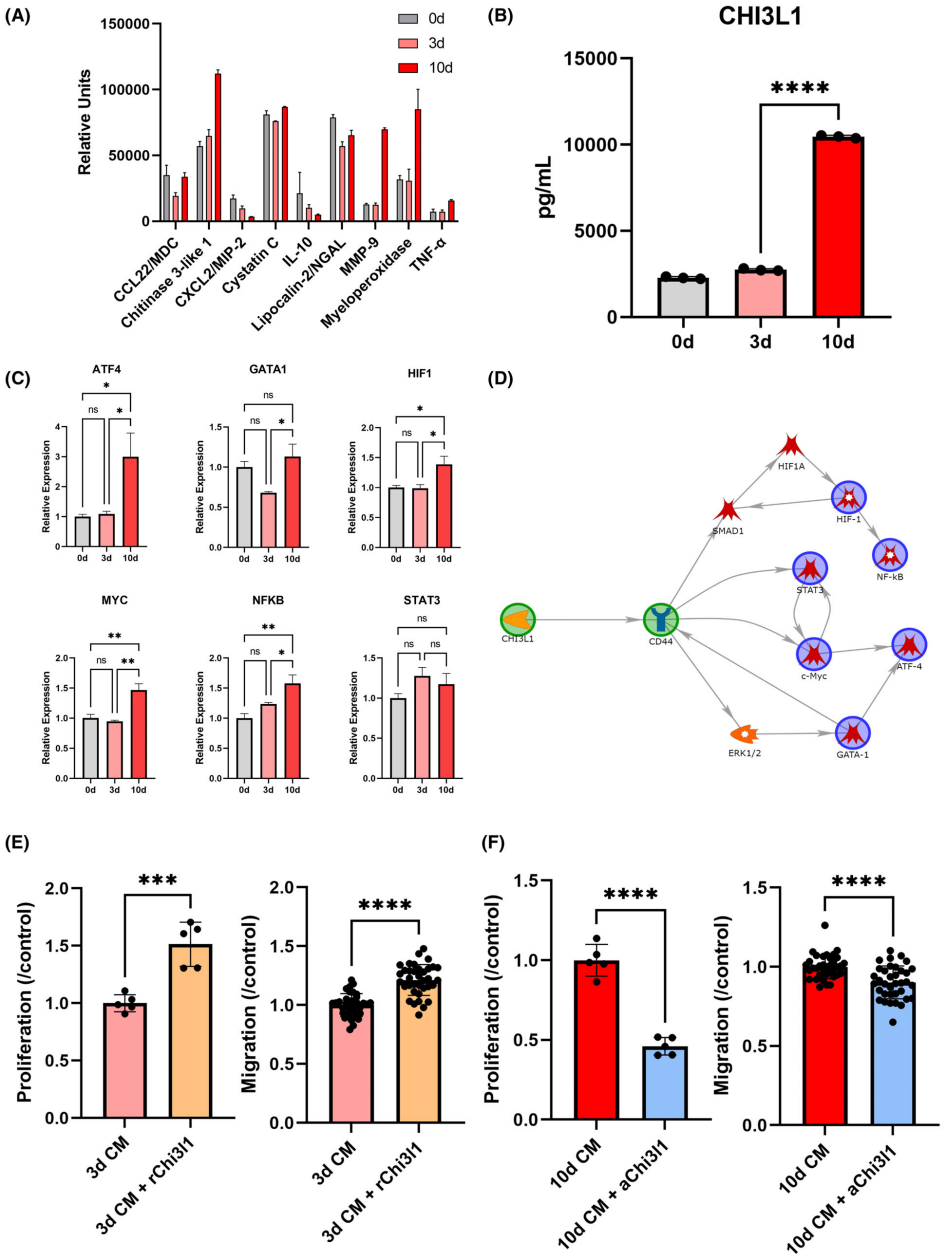
Gr1+ cells in the early metastatic lung drive oncogenic phenotypes in tumor cells through secretion of CHI3L1. (A) Protein secretion by Gr1+ cells broadly changes with disease progression with strong upregulation of CHI3L1. (B) Quantitative analysis by enzyme‐linked immunosorbent assay demonstrates a significant increase in CHI3L1 secretion by Gr1+ cells. (C) Oncogenic transcription factors are found to be upregulated in 4T1 cells in metastatic Gr1+ cell‐conditioned media when comparing conditioned media from 0 days (0d), 3 days (3d), and 10 days after inoculation (10d). (D) Pathway analysis identifies that upregulated transcription factors are directly downstream of CHI3L1 signaling through binding to CD44. The addition of recombinant CHI3L1 to 3 days conditioned media increases tumor cell proliferation and migration (E), while the blocking of CHI3L1 via anti‐CHI3L1 (aChi3l1) in 10 days conditioned media inhibits proliferation and migration (F) in *in vitro* assays. Two‐tailed unpaired *t*‐tests assuming unequal variance were performed for single comparisons between two conditions, *P ≤ 0.05, **P ≤ 0.01, ***P ≤ 0.001, ****P ≤ 0.0001; ns, no significance. Bars indicate mean ± standard deviation with *n* = 3 technical replicates for each assay in (B) and (C) and at least *n* = 5 technical replicates for assays in (E) and (F). Cells used to generate conditioned media were pooled from *n* = 5 biological replicates per condition.

Gr1+ myeloid cells support tumor cell colonization through multiple mechanisms, yet the specific signals that enable metastasis in the early metastatic niche, but not the premetastatic niche, are unknown [44]. Since Gr1+ cell‐conditioned media promoted 4T1 cell migration and proliferation, we hypothesize that Gr1+ cells secrete signals that directly promote oncogenic phenotypes in tumor cells. We characterized the secretome of 0, 3, and 10 days lungs through a protein array. Proteins such as CCL22, CHI3L1, MMP9, MPO, and TNFα were upregulated from 3 to 10 days conditioned media (Fig. 4A, Fig. S6A,B). Of the upregulated proteins, CHI3L1 was secreted at the highest levels relative to other proteins and exhibited the largest fold change (1.73‐fold) between 3 and 10 days lungs. To support the semiquantitative protein array, an ELISA was performed on Gr1+ cell‐conditioned media, and CHI3L1 was found to be secreted at 3.82‐fold higher levels by 10 days Gr1+ cells compared to the 3 days Gr1+ cells (Fig. 4B). CHI3L1 has previously been linked to increased metastasis and poor survival outcomes, suggesting that Gr1+ cell‐secreted CHI3L1 may play a significant role in the early metastatic niche [45].

To assess the influence of changes in CHI3L1 secretion on tumor cell phenotype, we measured transcription factor activity in 4T1 cells cultured in Gr1+ cell‐conditioned media. 4T1 cells were transfected with luminescent reporters for promoters corresponding to known oncogenic transcription factors, and transcription factor activity was monitored after 24 h. Seven of the nine assayed oncogenic transcription factors had upregulated activity in 4T1 cells cultured in 10 days Gr1+ cell‐conditioned media relative to 0 or 3 days Gr1+ cell‐conditioned media (Fig. 4C, Fig. S6C). Pathway analysis by metacore revealed that 5 of the 7 upregulated oncogenic transcription factors (ATF4, GATA1, HIF, MYC, NFκB, STAT3) are directly downstream of CHI3L1‐CD44 signaling, suggesting that CHI3L1 may contribute to oncogenic phenotypes induced by conditioned media (Fig. 4D). To verify that CHI3L1 was responsible for changes in tumor cell phenotype, recombinant CHI3L1 was added to 3 days Gr1+ cell‐conditioned media, and CHI3L1 in 10 days Gr1+ cell‐conditioned media was neutralized with anti‐CHI3L1. The addition of recombinant CHI3L1 to 3 days Gr1+ cell‐conditioned media increased tumor cell proliferation and migration (1.51‐fold and 1.20‐fold, respectively), while the addition of anti‐CHI3L1 to 10 days Gr1+ cell‐conditioned media reduced proliferation and migration (2.17‐fold and 1.16‐fold, respectively) (Fig. 4E,F). Overall, these data indicate that metastatic Gr1+ cells may promote tumor cell colonization through CHI3L1 signaling and that CHI3L1 can be targeted to inhibit oncogenic signaling.

### 3.5 Polymeric nanoparticles reduce granulocyte accumulation and inhibit CHI3L1 signaling

**Fig. 5:**
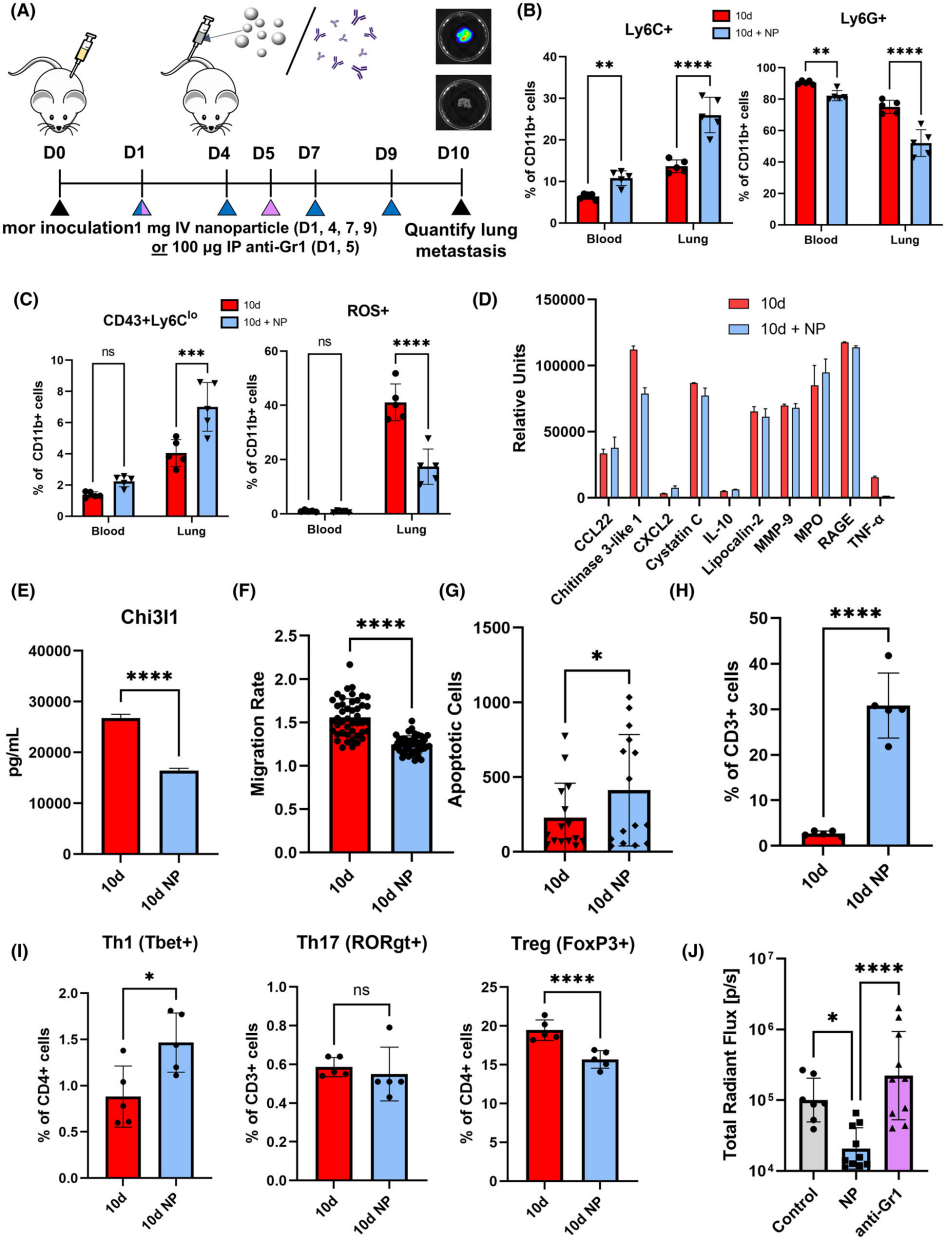
Poly (lactide‐co‐glycolide) nanoparticles (NP) alter myeloid cell composition of the lung and inhibit tumor‐supportive changes in Gr1+ cell phenotype. (A) Schematic of nanoparticle or anti‐Gr1 dosing. (B) Nanoparticles increase the proportion of Ly6C+ monocytes and decrease the proportion of Ly6G+ neutrophils in the lungs and blood of early metastatic mice relative to untreated mice at 10 days after inoculation (10d). (C) Nanoparticle treatment increases the proportion of nonclassical monocytes and reduces ROS‐expressing neutrophils in early metastatic mice relative to untreated mice. (D) Treatment with nanoparticles reduces levels of CHI3L1, but not other proteins that were upregulated with disease progression. (E) Nanoparticle treatment significantly reduces CHI3L1 secretion by Gr1+ cells. In coculture, lung Gr1+ cells from nanoparticle‐treated mice induce less tumor cell migration (F) and higher levels of tumor cell apoptosis (G) than Gr1+ cells from untreated mice. Gr1+ cells from nanoparticle‐treated lungs are less suppressive of T‐cell proliferation (H) and increase expression of T‐bet in CD4+ T cells (I) in coculture. (J) Nanoparticles inhibit metastasis and reduce metastatic burden relative to control, while anti‐Gr1 fails to control metastases as measured by luminescent signals from tumor cells. Two‐tailed unpaired *t*‐tests assuming unequal variance were performed for single comparisons between two conditions, *P ≤ 0.05, **P ≤ 0.01, ***P ≤ 0.001, ****P ≤ 0.0001; ns, no significance. Bars indicate mean ± standard deviation with *n* = 5 biological replicates for (B), (C), and (I) and *n* = 10 biological replicates for (J). At least *n* = 3 technical replicates were performed for assays in (E), (F), (G), and (H), with cells pooled from *n* = 5 biological replicates.

We hypothesize that we can target Gr1+ cells at the metastatic niche to inhibit CHI3L1 secretion and reduce tumor cell colonization. We have previously reported that cargo‐free, PLG nanoparticles alter immune cell trafficking and reduce tissue‐specific myeloid inflammation [22]. The nanoparticles are approximately 500 nm in diameter and possess a negative surface charge, such that the nanoparticles can associate with circulating myeloid cells (Fig. S7A). We intravenously injected nanoparticles into 4T1 tumor‐bearing mice on days 1, 4, 7, and 9 after inoculation (Fig. 5A). At 10 days after inoculation, nanoparticle treatment reduced the overall accumulation of myeloid cells and inhibited the shift towards tumor‐promoting, granulocytic phenotypes, skewing Gr1+ cells towards monocytes both in the lung and in circulating blood (Fig. 5B, Figs S7B and S8A). Nanoparticle treatment reduced the proportion of neutrophils in the lung and blood 1.44‐fold and 1.10‐fold, respectively, and increased the proportion of monocytes in the lung and blood 1.90‐fold and 1.69‐fold, respectively. Nanoparticles also reduced the proportion of ROS‐expressing neutrophils (1.28‐fold) and increased the proportion of CD43+ nonclassical monocytes (1.73‐fold) at the lung, effectively inhibiting changes in myeloid cell phenotypes seen between premetastatic and early metastatic lungs (Fig. 5C, Fig. S8B,C). CD4+ T cells in the lungs of nanoparticle‐treated mice trended towards higher proportions of T‐bet+ and RORγt+ cells (Figs S8D and S9).

The impact of nanoparticle internalization on Gr1+ cell phenotype was analyzed with single‐cell RNA sequencing, comparing Gr1+ cells that had internalized Cy5.5‐labeled nanoparticles (NP+) and Gr1+ cells that had not internalized nanoparticles (NP−). Compared to Gr1+ cells from untreated mice, we found that core neutrophil transcriptional programs were conserved between NP+ and NP− cells, with no upregulation of apoptotic or necrotic pathways attributed to nanoparticle uptake (Fig. S10). Pathway enrichment demonstrated that nanoparticle treatment reduces the expression of genes associated with JAK–STAT, interferon, and TGFβ signaling relative in Gr1+ cells from nanoparticle‐treated mice relative to Gr1+ cells from untreated mice. Interestingly, the downregulation of these signaling pathways was observed in both NP+ and NP− cells. Conversely, NP+ cells exhibited some unique transcriptional characteristics and downregulated genes associated with hypoxia, Kras signaling, Mtorc1 signaling, and P53 signaling, which was not observed in NP− cells.

We next investigated whether nanoparticle treatment inhibits the acquisition of tumor‐supportive functions by Gr1+ cells in the lung. Nanoparticle treatment significantly reduced the secretion of CHI3L1 by 10 days lung Gr1+ cells, with a 1.63‐fold decrease relative to untreated mice (Fig. 5D,E, Fig. S11). Interestingly, nanoparticles did not meaningfully affect the secretion of other proteins that were upregulated in early metastatic lungs, suggesting that therapeutic effects are driven by changes in CHI3L1 secretion or direct cell–cell interactions. Gr1+ cells from the lungs of nanoparticle‐treated mice induced more tumor cell apoptosis and less migration compared to Gr1+ cells from untreated mice (1.80‐fold increase and 1.25‐fold decrease, respectively) (Fig. 5F,G). Furthermore, Gr1+ cells from nanoparticle‐treated mice were less suppressive of T‐cell proliferation and function. T cells cocultured with Gr1+ cells from nanoparticle‐treated mice were more proliferative (11.5‐fold increase) and expressed higher levels of T‐bet (1.66‐fold increase) (Fig. 5H,I). While the expression of RORγt did not change, Gr1+ cells from nanoparticle‐treated mice reduced the expression of FoxP3 (1.24‐fold decrease). Collectively, these data demonstrate that nanoparticles reprogram Gr1+ cells and inhibit the acquisition of tumor‐supportive phenotypes associated with an early metastatic niche.

We hypothesize that nanoparticle modulation of Gr1+ cell phenotype disrupts the metastatic niche and inhibits tumor cell colonization. Nanoparticle treatment significantly reduced metastatic lesions in the lung compared to untreated mice (4.82‐fold reduction), and fewer nanoparticle‐treated mice had detectable metastases (2/10 mice) compared to untreated mice (6/7 mice) (Fig. 5J, Fig. S12A). Importantly, depletion of Gr1+ cells with anti‐Gr1 did not reduce the metastatic burden or the incidence of metastasis (8/10 mice). Although anti‐Gr1 was effective at depleting Gr1+ cells from the lung, this did not reduce the metastatic burden at the lung (Fig. S11B). Collectively, these data indicate that the modulation of myeloid cell accumulation and phenotype by nanoparticles, but not antibody‐based depletion, inhibits metastasis and demonstrates the potential of nanoparticles as an immunotherapy for disrupting the metastatic niche.

## 4 Discussion

Myeloid cells undergo a critical transformation in phenotype between the premetastatic and early metastatic stages of disease to enable tumor cell colonization. In addition to the systemic dysregulation of myeloid cell phenotype, myeloid cells that infiltrate tissues interact with resident stromal cells can acquire tissue‐specific, tumor‐supportive phenotypes that are reported to drive immunosuppression and enable cancer metastasis [46, 47]. While the suppression of T and natural killer cell function by myeloid cells is well‐studied, direct interactions between tumor cells and myeloid cells are thought to be especially important in the early metastatic niche [48]. Tumor‐supportive myeloid cell subsets that directly promote tumor cell colonization exist alongside tumor‐suppressive subsets that drive antitumor immunity. A result of these competing phenotypes is that treatments that nonspecifically target myeloid cells have shown limited efficacy in preventing metastasis [12]. The identification of specific myeloid cell phenotypes that distinguish premetastatic tissue from metastatic tissue could enable the targeted treatment of signaling mechanisms that drive the early seeding of tumor cells. In this study, we find that Gr1+ myeloid cells transition towards tumor‐supportive, granulocytic subsets that support tumor cell expansion and suppress T‐cell immunity, in part, through the secretion of CHI3L1. Furthermore, we report that treatment with polymer nanoparticles reduced the accumulation of neutrophils, downregulated CHI3L1 secretion, and shifted Gr1+ cells towards antitumor phenotypes, demonstrating the potential of nanoparticles to inhibit maturation of the metastatic niche.

Cancer progression is characterized by increasing immune dysregulation, and we observed that the early metastatic niche is characterized by the accumulation of tumor‐supportive, granulocytic myeloid cells. Whereas healthy and premetastatic lungs were primarily composed of nonclassical CD43+Ly6C^lo^ monocytes, the early metastatic niche at 10 days was characterized by the influx of neutrophils and classical CD43‐Ly6C^hi^ monocytes. Notably, while the absolute number of nonclassical monocytes increased, the influx of neutrophils and classical monocytes was fivefold greater, overwhelming these potentially antitumor subsets. Nonclassical monocytes have antitumor roles and promote the recruitment and activation of T and natural cells, while classical monocytes are associated with tumor‐associated macrophages and T‐cell suppression [36, 49]. Similarly, elevated levels of granulocytic myeloid cells are clinically associated with metastatic disease and dysregulated neutrophils are known to promote tumor cell colonization and immune suppression [50]. While granulocytes are thought to be less immunosuppressive than monocytes on a per‐cell basis, the influx of granulocytes and net increase in myeloid cells with disease progression is thought to drive an immunosuppressive environment [51, 52]. Accordingly, while we observed a modest increase in neutrophil accumulation in premetastatic lungs, the metastatic niche did not acquire tumor‐promoting and immunosuppressive capacities until the early metastatic stage of disease. Gr1+ cells from metastatic lungs, but not premetastatic lungs, expressed elevated levels of ROS, which suppress T‐cell function [53]. Accordingly, Gr1+ cells from metastatic lungs were more suppressive of T‐cell proliferation and cytotoxicity compared to Gr1+ cells from premetastatic lungs. These functional changes support previous transcriptomic analyses suggesting that myeloid cells in an established metastatic niche are phenotypically distinct from myeloid cells in a premetastatic niche or in healthy tissue [13, 54]. Importantly, changes in myeloid cell composition in the lung were not entirely driven by systemic immune dysregulation. The upregulation of ROS was observed in neutrophils in the lung, but not in the blood. These findings are consistent with previous reports that recruited myeloid cells acquire immunosuppressive phenotypes through interactions with stromal cells after infiltrating the lung [47]. Collectively, these findings demonstrate that the maturation of a metastatic niche is characterized by the accumulation of tumor‐supportive myeloid cell subsets.

Myeloid cells create a highly inflammatory environment at the metastatic niche and secrete cytokines that promote tumor cell recruitment and survival. We found that the increased migration and proliferation of tumor cells induced by 10 days Gr1+ cells in coculture was conserved in Gr1+ cell‐conditioned media, suggesting that these functions are linked to secreted factors. While the secretome of Gr1+ cells broadly changes between the premetastatic and early metastatic niche, we found that increased tumor cell proliferation and migration were linked to the secretion of CHI3L1. CHI3L1 is known to promote oncogenic phenotypes in tumor cells and has been reported to promote metastasis in various models of cancer, including lung cancer and melanoma [55, 56]. The neutralization of CHI3L1 *in vivo* with anti‐CHI3L1 antibodies or soluble chitin has been found to inhibit lung metastasis and induce antitumor immune responses [57, 58, 59]. Consistent with these reports, we found that the *in vitro* neutralization of CHI3L1 with NPs in the 4T1 model of triple‐negative breast cancer directly reduces tumor cell migration and proliferation and directly inhibits the tumor‐supportive functions of Gr1+ cells. CHI3L1 is known to directly interact with tumor cells by binding to the CD44 receptor [60]. Accordingly, we found that the transcription factors MYC, GATA1, ATF4, HIF1, and NFκB, which are directly activated by CD44 signaling, were upregulated in response to Gr1+ cell‐conditioned media [61, 62, 63]. While we found that CHI3L1 directly promotes tumor cell proliferation and migration, CHI3L1 is also associated with the expression of other oncogenic factors such as MMP9, which was strongly expressed in the early metastatic niche in our present study [59]. Interestingly, while nanoparticle treatment reduced CHI3L1 expression and tumor‐supportive functions in Gr1+ cells, nanoparticle treatment was not associated with reduced expression of MMP9 or other associated oncogenic factors. The inhibition of metastasis *in vivo* despite the continued expression of MMP9 indicates that factors other than CHI3l1 may not be critical to tumor cell colonization at this stage. Overall, these data show that metastatic Gr1+ cells acquire tumor‐supportive functions that distinguish them from premetastatic Gr1+ cells and that these functions are mediated, in part, by the upregulation of CHI3L1.

We found that cargo‐free, PLG nanoparticles inhibited maturation of the metastatic niche and inhibited tumor‐supportive phenotypic shifts in Gr1+ cells. Intravenously injected nanoparticles associate with immune cells in circulation and have been found to alter the trafficking and phenotype of myeloid cells in tissues [22, 64, 65, 66]. Nanoparticles were found to reduce disease‐associated inflammation and influence myeloid cell phenotypes in a manner based on nanoparticle composition [67]. Consistent with these reports, we found that nanoparticles altered myeloid cell composition at the lung, inhibiting the accumulation of granulocytic and tumor‐supportive subsets at the early metastatic niche. While systemic depletion of myeloid cells using anti‐Gr1 has been reported to reduce tumor cell colonization with advanced metastases, we found that anti‐Gr1 was not therapeutically effective during the premetastatic stage of disease [68, 69]. The depletion of Gr1+ cells in the premetastatic stage of disease has been found to increase metastatic burden and shorten survival [14]. Since Gr1+ cells possess antitumor functions during this stage, depletion may accelerate the replacement of these cells with increasingly dysregulated and tumor‐supportive cells [14]. Rather than depleting myeloid cells, nanoparticles are hypothesized to alter trafficking and reprogram cell phenotypes and do not affect existing myeloid populations at the lung [64]. Via single‐cell RNA sequencing, we demonstrate that nanoparticle treatment broadly influenced gene expression in Gr1+ cells and downregulated tumor‐promoting pathways such as mTOR and KRAS signaling, which have previously been linked to myeloid‐derived suppressor cell‐like phenotypes in neutrophils and neutrophil recruitment, respectively [70, 71]. Interestingly, nanoparticle treatment was also found to downregulate inflammatory pathways in NP− cells, suggesting that while the reprogramming of transcriptional profiles is driven by nanoparticle treatment, the effect is not restricted to cells that have internalized nanoparticles. Importantly, nanoparticle treatment was not found to upregulate apoptotic pathways or reduce cell viability, indicating that nanoparticles operate in a mechanism distinct from depletion. Nanoparticle treatment was able to inhibit metastasis *in vivo* and maintained antitumor Gr1+ cell phenotypes. Gr1+ cells in nanoparticle‐treated mice induced higher levels of tumor cell apoptosis and were less suppressive of T‐cell proliferation compared to Gr1+ cells in untreated mice. Importantly, the nanoparticles developed for the present study are not loaded with a drug or active agent, and immune cell reprogramming is based on the intrinsic immunomodulatory properties of the particles. While chitin nanoparticles have previously been developed to target myeloid cell‐derived CHI3L1, we were able to reduce CHI3L1 secretion and inhibit metastasis using cargo‐free, PLG nanoparticles [56]. The PLG nanoparticles used in the present study did not specifically target CHI3L1, but instead altered myeloid cell composition and phenotypes at the lung, which led to the downregulation of CHI3L1. Immune modulation by cargo‐free nanoparticles is controlled by the formulation and composition of the particles, motivating further investigation into the mechanisms of nanoparticle reprogramming of cancer‐dysregulated myeloid cells [67].

## 5 Conclusion

In conclusion, our data demonstrate that maturation of the metastatic niche is driven by the tissue‐specific accumulation of tumor‐supportive neutrophils. Gr1+ cells with antitumor functions are replaced by neutrophils directly promote tumor cell colonization, in part, through the secretion of CHI3L1. Reprogramming by cargo‐free, PLG nanoparticles inhibited oncogenic shifts in phenotype and inhibited metastasis, demonstrating the potency of nanoparticle immunomodulation and the significance of myeloid cells in early metastasis. Therapies that specifically target myeloid cells, yet broadly reprogram myeloid cell phenotype, may more effectively inhibit the development of the metastatic niche in comparison to therapies that modulate specific pathways. Further investigation into the reprogramming of myeloid cells with nanoparticles to achieve desired phenotypes will inform future therapies for relieving immunosuppression and treating metastasis.

## Conflict of interest

LDS consultants and has financial interests in Cour Pharmaceutical Development Company, Inc., which licenses the nanoparticle technology used here that is described in patent US‐20150190485. All other authors declare they have no competing interests.

## Author contributions

JAM, SMO, JSJ, and LDS contributed to the conceptualization, writing, and editing of the manuscript. JAM, SMO, YZ, RSP, KK, KVG, IAS, and GE contributed to the methodology and investigation. JSJ and LDS contributed to the acquisition of funding.

## Ethics statement

All animal work was approved by the University of Michigan Institutional Animal Care & Use Committee under protocols #00009715 and #00011457.

## Peer review

The peer review history for this article is available at https://www.webofscience.com/api/gateway/wos/peer‐review/10.1002/1878‐0261.70040.

## Supporting information


**Fig. S1.** Gating scheme for flow cytometric analysis.

**Fig. S2.** Representative images for flow cytometric analysis. (A) Representative images for Ly6C+/Ly6G+ populations among CD11b+ cells, corresponding to Fig. 1B. (B) Representative images for CD43+ populations among Ly6C+ cells, and reactive oxygen species (ROS) + populations among LyG6+ cells, corresponding to Fig. 1C. (C) Representative images for Tbet+ and RORγt+ populations among CD4+ cells, corresponding to Fig. 1E.

**Fig. S3.** Absolute cell counts and gene expression of Gr1+ cells at 0 days (0d), 3 days (3d), and 10 days after inoculation (10d). (A) The number of classical monocytes per mg of lung tissue increases to higher levels than those of nonclassical monocytes. (B) Neutrophil‐associated genes are upregulated in Gr1+ cells from metastatic lungs compared to Gr1+ cells from premetastatic lungs. Two‐tailed unpaired *t*‐tests assuming unequal variance were performed for single comparisons between two conditions, **P ≤ 0.01, ***P ≤ 0.001. Bars indicate mean ± standard deviation with *n* = 5 biological replicates.

**Fig. S4.** Representative images for flow cytometric analysis. (A) Representative images for Ly6C+/Ly6G+ populations among CD11b+ cells, corresponding to Fig. 2A. (B) Representative images for CD43+ populations among Ly6C+ cells, and reactive oxygen‐species expressing‐populations (dihydrorhodamine – DHR+) among LyG6+ cells, corresponding to Fig. 2C.

**Fig. S5.** Gr1+ cell conditioned media from 0 days (0d), 3 days (3d), and 10 days after inoculation (10d) does not significantly influence T cell proliferation. Two‐tailed unpaired *t*‐tests assuming unequal variance were performed for single comparisons between two conditions, ns – no significance. Bars indicate mean ± standard deviation with *n* = 5 technical replicates.

**Fig. S6.** Secreted factors by Gr1+ cells modulate transcription factor activity in 4T1 cells. (A) Full panel of immunoassay proteins identified as highly secreted (> 5000 relative units). (B) Raw protein membrane array images. (C) Gr1+ cell conditioned media from 0 days (0d), 3 days (3d), and 10 days after inoculation (10d) altered activity of transcription factors that were not directly related to Chi3l1 signaling. Two‐tailed unpaired *t*‐tests assuming unequal variance were performed for single comparisons between two conditions, *P ≤ 0.05, **P ≤ 0.01.

**Fig. S7.** Nanoparticles (NP) reduce immune accumulation at the lung. (A) Nanoparticles are approximately 500 nm in diameter and have a negative surface charge. (B) Nanoparticles reduce the accumulation of myeloid cells, especially of neutrophils and classical monocytes compared to untreated mice 10 days after inoculation (10d). Two‐tailed unpaired *t*‐tests assuming unequal variance were performed for single comparisons between two conditions, ****P ≤ 0.0001. Bars indicate mean ± standard deviation with *n* = 5 biological replicates.

**Fig. S8.** Representative images for flow cytometric analysis. (A) Representative images for Ly6C+/Ly6G+ populations among CD11b+ cells between untreated (10d) and nanoparticle‐treated mice (10d + NP), corresponding to Fig. 5B. (B) Representative images for CD43+ populations among Ly6C+ cells, corresponding to Fig. 5C. (C) Representative images for reactive oxygen species (ROS) + populations among Ly6G+ cells, corresponding to Fig. 5C. (D) Representative images for Tbet+ and RORγt+ populations among CD4+ cells, corresponding to Fig. S7B.

**Fig. S9.** Nanoparticles (NP) drive a trend towards increased expression of Tbet and RORγt in CD4+ T cells *in vivo* in comparison to untreated mice 10 days after inoculation (10d). Two‐tailed unpaired *t*‐tests assuming unequal variance were performed for single comparisons between two conditions. Bars indicate mean ± standard deviation with *n* = 5 biological replicates.

**Fig. S10.** Single‐cell RNA sequencing demonstrates that nanoparticles broadly reprogram Gr1+ cells relative to cells in untreated mice. Nanoparticle treatment downregulates genes associated with JAK–STAT, interferon, and TGFβ signaling in both nanoparticle (NP)+ and NP− cells. The influence of nanoparticles on hypoxia, Kras, Mtorc1, and P53 is restricted to NP+ cells.

**Fig. S11.** Full panel of immunoassay proteins identified as highly secreted in nanoparticle‐treated mice (> 5000 relative units).

**Fig. S12.** Nanoparticles (NP), but not anti‐Gr1, inhibit lung metastasis. (A) Nanoparticles, but not anti‐Gr1, reduce the incidence of lung metastasis. (B) Anti‐Gr1 treatment reduces Gr1 cells to levels undetectable by flow cytometry in the lung, blood, and spleen. Two‐tailed unpaired *t*‐tests assuming unequal variance were performed for single comparisons between two conditions. Bars indicate mean ± standard deviation with *n* = 3 biological replicates.

## Data Availability

Additional data that support the findings of this study are available from the corresponding author upon request.

## References

[1] Siegel RL , Giaquinto AN , Jemal A . Cancer statistics, 2024. CA Cancer J Clin. 2024;74(1):12–49. 10.3322/caac.21820 38230766

[2] Dillekås H , Rogers MS , Straume O . Are 90% of deaths from cancer caused by metastases? Cancer Med. 2019;8(12):5574–5576. 10.1002/cam4.2474 31397113 PMC6745820

[3] Chaffer CL , Weinberg RA . A perspective on cancer cell metastasis. Science. 2011;331(6024):1559–1564. 10.1126/science.1203543 21436443

[4] Debien V , De Caluwé A , Wang X , Piccart‐Gebhart M , Tuohy VK , Romano E , et al. Immunotherapy in breast cancer: an overview of current strategies and perspectives. NPJ Breast Cancer. 2023;9(1):7. 10.1038/s41523-023-00508-3 36781869 PMC9925769

[5] Celià‐Terrassa T , Kang Y . Metastatic niche functions and therapeutic opportunities. Nat Cell Biol. 2018;20(8):868–877. 10.1038/s41556-018-0145-9 30050120

[6] Groth C , Hu X , Weber R , Fleming V , Altevogt P , Utikal J , et al. Immunosuppression mediated by myeloid‐derived suppressor cells (MDSCs) during tumour progression. Br J Cancer. 2019;120(1):16–25. 10.1038/s41416-018-0333-1 30413826 PMC6325125

[7] Gabrilovich DI . Myeloid‐derived suppressor cells. Cancer Immunol Res. 2017;5(1):3–8. 10.1158/2326-6066.CIR-16-0297 28052991 PMC5426480

[8] Yan HH , Pickup M , Pang Y , Gorska AE , Li Z , Chytil A , et al. Gr‐1+CD11b+ myeloid cells tip the balance of immune protection to tumor promotion in the premetastatic lung. Cancer Res. 2010;70(15):6139–6149. 10.1158/0008-5472.CAN-10-0706 20631080 PMC4675145

[9] Li R , Salehi‐Rad R , Crosson W , Momcilovic M , Lim RJ , Ong SL , et al. Inhibition of granulocytic myeloid‐derived suppressor cells overcomes resistance to immune checkpoint inhibition in LKB1‐deficient non‐small cell lung cancer. Cancer Res. 2021;81(12):3295–3308. 10.1158/0008-5472.CAN-20-3564 33853830 PMC8776246

[10] Wang J , Ocadiz‐Ruiz R , Hall MS , Bushnell GG , Orbach SM , Decker JT , et al. A synthetic metastatic niche reveals antitumor neutrophils drive breast cancer metastatic dormancy in the lungs. Nat Commun. 2023;14(1):4790. 10.1038/s41467-023-40478-5 37553342 PMC10409732

[11] Bronte V , Brandau S , Chen SH , Colombo MP , Frey AB , Greten TF , et al. Recommendations for myeloid‐derived suppressor cell nomenclature and characterization standards. Nat Commun. 2016;7:12150. 10.1038/ncomms12150 27381735 PMC4935811

[12] Veglia F , Sanseviero E , Gabrilovich DI . Myeloid‐derived suppressor cells in the era of increasing myeloid cell diversity. Nat Rev Immunol. 2021;21(8):485–498. 10.1038/s41577-020-00490-y 33526920 PMC7849958

[13] Kaczanowska S , Beury DW , Gopalan V , Tycko AK , Qin H , Clements ME , et al. Genetically engineered myeloid cells rebalance the core immune suppression program in metastasis. Cell. 2021;184(8):1–20. 10.1016/j.cell.2021.02.048 33765443 PMC8344805

[14] Orbach SM , Brooks MD , Zhang Y , Campit SE , Bushnell GG , Decker JT , et al. Single‐cell RNA‐sequencing identifies anti‐cancer immune phenotypes in the early lung metastatic niche during breast cancer. Clin Exp Metastasis. 2022;39(6):865–881. 10.1007/s10585-022-10185-4 36002598 PMC9643644

[15] McGinnis CS , Miao Z , Superville D , Yao W , Goga A , Reticker‐Flynn NE , et al. The temporal progression of lung immune remodeling during breast cancer metastasis. Cancer Cell. 2024;42(6):1018–1031. 10.1016/j.ccell.2024.05.004 38821060 PMC11255555

[16] Cassetta L , Pollard JW . A timeline of tumour‐associated macrophage biology. Nat Rev Cancer. 2023;23(4):238–257. 10.1038/s41568-022-00547-1 36792751

[17] Qian BZ , Li J , Zhang H , Kitamura T , Zhang J , Campion LR , et al. CCL2 recruits inflammatory monocytes to facilitate breast‐tumour metastasis. Nature. 2011;475(7355):222–225. 10.1038/nature10138 21654748 PMC3208506

[18] Cassetta L , Pollard JW . Targeting macrophages: therapeutic approaches in cancer. Nat Rev Drug Discov. 2018;17(12):887–904. 10.1038/nrd.2018.169 30361552

[19] Bosiljcic M , Cederberg RA , Hamilton MJ , LePard NE , Harbourne BT , Collier JL , et al. Targeting myeloid‐derived suppressor cells in combination with primary mammary tumor resection reduces metastatic growth in the lungs. Breast Cancer Res. 2019;21:103. 31488209 10.1186/s13058-019-1189-x PMC6727565

[20] Kamran N , Kadiyala P , Saxena M , Candolfi M , Li Y , Moreno‐Ayala MA , et al. Immunosuppressive myeloid cells' blockade in the glioma microenvironment enhances the efficacy of immune‐stimulatory gene therapy. Mol Ther. 2017;25(1):232–248. 10.1016/j.ymthe.2016.10.003 28129117 PMC5363306

[21] Zhang Y , Hughes KR , Raghani RM , Ma J , Orbach S , Jeruss JS , et al. Cargo‐free immunomodulatory nanoparticles combined with anti‐PD‐1 antibody for treating metastatic breast cancer. Biomaterials. 2021;269:120666. 10.1016/j.biomaterials.2021.120666 33461057 PMC7870582

[22] Raghani RM , Ma JA , Zhang Y , Orbach SM , Wang J , Zeinali M , et al. Myeloid cell reprogramming alleviates immunosuppression and promotes clearance of metastatic lesions. Front Oncol. 2022;12:1039993. 10.3389/fonc.2022.1039993 36479083 PMC9720131

[23] Hunter Z , McCarthy DP , Yap WT , Harp CT , Getts DR , Shea LD , et al. A biodegradable nanoparticle platform for the induction of antigen‐specific immune tolerance for treatment of autoimmune disease. ACS Nano. 2014;8(3):2148–2160. 10.1021/nn405033r 24559284 PMC3990004

[24] Love MI , Huber W , Anders S . Moderated estimation of fold change and dispersion for RNA‐seq data with DESeq2. Genome Biol. 2014;15(12):1–21. 10.1186/s13059-014-0550-8 PMC4302049 25516281

[25] Subramanian A , Tamayo P , Mootha VK , Mukherjee S , Ebert BL , Gillette MA , et al. Gene set enrichment analysis: a knowledge‐based approach for interpreting genome‐wide expression profiles. Proc Natl Acad Sci USA. 2005;102(43):15545–15550. 10.1073/pnas.0506580102 16199517 PMC1239896

[26] Young MD , Behjati S . SoupX removes ambient RNA contamination from droplet‐based single‐cell RNA sequencing data. Gigascience. 2020;9(12):1–10. 10.1093/gigascience/giaa151 PMC7763177 33367645

[27] Subramanian A , Alperovich M , Yang Y , Li B . Biology‐inspired data‐driven quality control for scientific discovery in single‐cell transcriptomics. Genome Biol. 2022;23(1):1–27. 10.1186/s13059-022-02820-w 36575523 PMC9793662

[28] McGinnis CS , Murrow LM , Gartner ZJ . DoubletFinder: doublet detection in single‐cell RNA sequencing data using artificial nearest neighbors. Cell Syst. 2019;8(4):329–337. 10.1016/j.cels.2019.03.003 30954475 PMC6853612

[29] Hafemeister C , Satija R . Normalization and variance stabilization of single‐cell RNA‐seq data using regularized negative binomial regression. Genome Biol. 2019;20(1):1–15. 10.1186/s13059-019-1874-1 31870423 PMC6927181

[30] Hao Y , Stuart T , Kowalski MH , Choudhary S , Hoffman P , Hartman A , et al. Dictionary learning for integrative, multimodal and scalable single‐cell analysis. Nat Biotechnol. 2024;42(2):293–304. 10.1038/s41587-023-01767-y 37231261 PMC10928517

[31] Mootha VK , Lindgren CM , Eriksson KF , Subramanian A , Sihag S , Lehar J , et al. PGC‐1α‐responsive genes involved in oxidative phosphorylation are coordinately downregulated in human diabetes. Nat Genet. 2003;34(3):267–273. 12808457 10.1038/ng1180

[32] Federico A , Monti S . HypeR: an R package for geneset enrichment workflows. Bioinformatics. 2020;36(4):1307–1308. 10.1093/bioinformatics/btz700 31498385 PMC7998712

[33] Schindelin J , Arganda‐Carreras I , Frise E , Kaynig V , Longair M , Pietzsch T , et al. Fiji: an open‐source platform for biological‐image analysis. Nat Methods. 2012;9(7):676–682. 10.1038/nmeth.2019 22743772 PMC3855844

[34] Weiss MS , Peñalver Bernabé B , Shin S , Asztalos S , Dubbury SJ , Mui MD , et al. Dynamic transcription factor activity and networks during ErbB2 breast oncogenesis and targeted therapy. Integr Biol. 2014;6(12):1170–1182. 10.1039/c4ib00086b PMC4237672 25303361

[35] Decker JT , Hobson EC , Zhang Y , Shin S , Thomas AL , Jeruss JS , et al. Systems analysis of dynamic transcription factor activity identifies targets for treatment in Olaparib resistant cancer cells. Biotechnol Bioeng. 2017;114(9):2085–2095. 10.1002/bit.26293 28322442 PMC5522358

[36] Robinson A , Han CZ , Glass CK , Pollard JW . Monocyte regulation in homeostasis and malignancy. Trends Immunol. 2021;42(2):104–119. 10.1016/j.it.2020.12.001 33446416 PMC7877795

[37] Gabrilovich DI , Ostrand‐Rosenberg S , Bronte V . Coordinated regulation of myeloid cells by tumours. Nat Rev Immunol. 2012;12(4):253–268. 10.1038/nri3175 22437938 PMC3587148

[38] Ostrand‐Rosenberg S , Fenselau C . Myeloid‐derived suppressor cells: immune‐suppressive cells that impair antitumor immunity and are sculpted by their environment. J Immunol. 2018;200(2):422–431. 10.4049/jimmunol.1701019 29311384 PMC5765878

[39] Zhong J , Li Q , Luo H , Holmdahl R . Neutrophil‐derived reactive oxygen species promote tumor colonization. Commun Biol. 2021;4(1):4–10. 10.1038/s42003-021-02376-8 34257370 PMC8277858

[40] Grieshaber‐Bouyer R , Radtke FA , Cunin P , Stifano G , Levescot A , Vijaykumar B , et al. The neutrotime transcriptional signature defines a single continuum of neutrophils across biological compartments. Nat Commun. 2021;12(1):1–21. 10.1038/s41467-021-22973-9 34001893 PMC8129206

[41] Andreu‐Sanz D , Kobold S . Role and potential of different T helper cell subsets in adoptive cell therapy. Cancers (Basel). 2023;15(6):1650. 10.3390/cancers15061650 36980536 PMC10046829

[42] Jin X , Mu P . Targeting breast cancer metastasis. Breast Cancer. 2015;9(S1):23–34. 10.4137/BCBCR.S25460 26380552 PMC4559199

[43] Fidler IJ . The pathogenesis of cancer metastasis: the “seed and soil” hypothesis revisited. Nat Rev Cancer. 2003;3:453–458. 10.1097/00006454-199103000-00027 12778135

[44] Kitamura T , Qian BZ , Pollard JW . Immune cell promotion of metastasis. Nat Rev Immunol. 2015;15(2):73–86. 10.1038/nri3789 25614318 PMC4470277

[45] Libreros S , Iragavarapu‐Charyulu V . YKL‐40/CHI3L1 drives inflammation on the road of tumor progression. J Leukoc Biol. 2015;98(6):931–936. 10.1189/jlb.3vmr0415-142r 26310833 PMC6608021

[46] Gong Z , Li Q , Shi J , Wei J , Li P , Chang CH , et al. Lung fibroblasts facilitate pre‐metastatic niche formation by remodeling the local immune microenvironment. Immunity. 2022;55(8):1483–1500.e9. 10.1016/j.immuni.2022.07.001 35908547 PMC9830653

[47] Gong Z , Li Q , Shi J , Li P , Hua L , Shultz LD , et al. Immunosuppressive reprogramming of neutrophils by lung mesenchymal cells promotes breast cancer metastasis. Sci Immunol. 2023;8(80):eadd5204. 10.1126/sciimmunol.add5204 36800412 PMC10067025

[48] Jia J , Wang Y , Li M , Wang F , Peng Y , Hu J , et al. Neutrophils in the premetastatic niche: key functions and therapeutic directions. Mol Cancer. 2024;23(1):200. 10.1186/s12943-024-02107-7 39277750 PMC11401288

[49] Olingy CE , Dinh HQ , Hedrick CC . Monocyte heterogeneity and functions in cancer. J Leukoc Biol. 2019;106(2):309–322. 10.1002/JLB.4RI0818-311R 30776148 PMC6658332

[50] Xiong S , Dong L , Cheng L . Neutrophils in cancer carcinogenesis and metastasis. J Hematol Oncol. 2021;14(1):1–17. 10.1186/s13045-021-01187-y 34674757 PMC8529570

[51] Kitamura T , Doughty‐Shenton D , Cassetta L , Fragkogianni S , Brownlie D , Kato Y , et al. Monocytes differentiate to immune suppressive precursors of metastasis‐associated macrophages in mouse models of metastatic breast cancer. Front Immunol. 2017;8:2004. 10.3389/fimmu.2017.02004 29387063 PMC5776392

[52] Movahedi K , Guilliams M , Van Den Bossche J , Van den Bergh R , Gysemans C , Beschin A , et al. Identification of discrete tumor‐induced myeloid‐derived suppressor cell subpopulations with distinct T cell suppressive activity. Blood. 2008;111(8):4233–4244. 10.1182/blood-2007-07-099226 18272812

[53] Wu M , Ma M , Tan Z , Zheng H , Liu X . Neutrophil: a new player in metastatic cancers. Front Immunol. 2020;11:1–14. 10.3389/fimmu.2020.565165 33101283 PMC7546851

[54] Alshetaiwi H , Pervolarakis N , McIntyre LL , Ma D , Nguyen Q , Rath JA , et al. Defining the emergence of myeloid‐derived suppressor cells in breast cancer using single‐cell transcriptomics. Sci Immunol. 2020;5(44):eaay6017. 10.1126/sciimmunol.aay6017 32086381 PMC7219211

[55] Zhao T , Su Z , Li Y , Zhang X , You Q . Chitinase‐3 like‐protein‐1 function and its role in diseases. Signal Transduct Target Ther. 2020;5(1):1–20. 10.1038/s41392-020-00303-7 32929074 PMC7490424

[56] Libreros S , Garcia‐Areas R , Keating P , Carrio R , Iragavarapu‐Charyulu VL . Exploring the role of CHI3L1 in “pre‐metastatic” lungs of mammary tumor‐bearing mice. Front Physiol. 2013;4:392. 10.3389/fphys.2013.00392 24399973 PMC3872303

[57] Ma B , Akosman B , Kamle S , Lee CM , He CH , Koo JS , et al. CHI3L1 regulates PD‐L1 and anti–CHI3L1–PD‐1 antibody elicits synergistic antitumor responses. J Clin Invest. 2021;131(21):e137750. 10.1172/JCI137750 34720089 PMC8553560

[58] Yu JE , Yeo IJ , Son DJ , Yun J , Han SB , Hong JT . Anti‐Chi3L1 antibody suppresses lung tumor growth and metastasis through inhibition of M2 polarization. Mol Oncol. 2022;16(11):2214–2234. 10.1002/1878-0261.13152 34861103 PMC9168758

[59] Libreros S , Garcia‐Areas R , Shibata Y , Carrio R , Torroella‐Kouri M , Iragavarapu‐Charyulu V . Induction of proinflammatory mediators by CHI3L1 is reduced by chitin treatment: decreased tumor metastasis in a breast cancer model. Int J Cancer. 2012;131(2):377–386. 10.1002/ijc.26379 21866546 PMC3288379

[60] Geng B , Pan J , Zhao T , Ji J , Zhang C , Che Y , et al. Chitinase 3‐like 1‐CD44 interaction promotes metastasis and epithelial‐to‐mesenchymal transition through β‐catenin/Erk/Akt signaling in gastric cancer. J Exp Clin Cancer Res. 2018;37(1):1–20. 10.1186/s13046-018-0876-2 30165890 PMC6117920

[61] Miyazono K , Maeda S , Imamura T . BMP receptor signaling: transcriptional targets, regulation of signals, and signaling cross‐talk. Cytokine Growth Factor Rev. 2005;16(3):251–263. 10.1016/j.cytogfr.2005.01.009 15871923

[62] Smith SM , Cai L . Cell specific CD44 expression in breast cancer requires the interaction of AP‐1 and NFκB with a novel cis‐element. PLoS One. 2012;7(11):e50867. 10.1371/journal.pone.0050867 23226410 PMC3511339

[63] Zhang Y , Xia H , Ge X , Chen Q , Yuan D , Chen Q , et al. CD44 acts through RhoA to regulate YAP signaling. Cell Signal. 2014;26(11):2504–2513. 10.1016/j.cellsig.2014.07.031 25101858

[64] Getts DR , Terry RL , Getts MT , Deffrasnes C , Müller M , van Vreden C , et al. Therapeutic inflammatory monocyte modulation using immune‐modifying microparticles. Sci Transl Med. 2014;6(219):219ra7. 10.1126/scitranslmed.3007563 PMC3973033 24431111

[65] Saito E , Kuo R , Pearson RM , Gohel N , Cheung B , King NJC , et al. Designing drug‐free biodegradable nanoparticles to modulate inflammatory monocytes and neutrophils for ameliorating inflammation. J Control Release. 2019;300:185–196. 10.1016/j.jconrel.2019.02.025 30822435 PMC6486651

[66] Park J , Zhang Y , Saito E , Gurczynski SJ , Moore BB , Cummings BJ , et al. Intravascular innate immune cells reprogrammed via intravenous nanoparticles to promote functional recovery after spinal cord injury. Proc Natl Acad Sci USA. 2019;116(30):14947–14954. 10.1073/pnas.1820276116 31285339 PMC6660718

[67] Casey LM , Kakade S , Decker JT , Rose JA , Deans K , Shea LD , et al. Cargo‐less nanoparticles program innate immune cell responses to toll‐like receptor activation. Biomaterials. 2019;218:119333. 10.1016/j.biomaterials.2019.119333 31301576 PMC6679939

[68] Fujita M , Kohanbash G , Fellows‐Mayle W , Hamilton RL , Komohara Y , Decker SA , et al. COX‐2 blockade suppresses gliomagenesis by inhibiting myeloid‐derived suppressor cells. Cancer Res. 2011;71(7):2664–2675. 10.1158/0008-5472.CAN-10-3055 21324923 PMC3075086

[69] Rose P , van den Engel NK , Kovács JR , Hatz RA , Boon L , Winter H . Anti‐gr‐1 antibody provides short‐term depletion of MDSC in lymphodepleted mice with active‐specific melanoma therapy. Vaccine. 2022;10(4):560. 10.3390/vaccines10040560 PMC9032646 35455309

[70] Mafi S , Mansoori B , Taeb S , Sadeghi H , Abbasi R , Cho WC , et al. mTOR‐mediated regulation of immune responses in cancer and tumor microenvironment. Front Immunol. 2022;12:774103. 10.3389/fimmu.2021.774103 35250965 PMC8894239

[71] Hamarsheh S , Groß O , Brummer T , Zeiser R . Immune modulatory effects of oncogenic KRAS in cancer. Nat Commun. 2020;11(1):5439. 10.1038/s41467-020-19288-6 33116132 PMC7595113

