# Supplementary figures and images for: Early metastasis is characterized by Gr1+ cell dysregulation and is inhibited by immunomodulatory nanoparticles
Source: Mol Oncol. 2025 Apr 23;19(10):2860–81. doi: 10.1002/1878-0261.70040 (PMC12515690; doi:10.1002/1878-0261.70040)

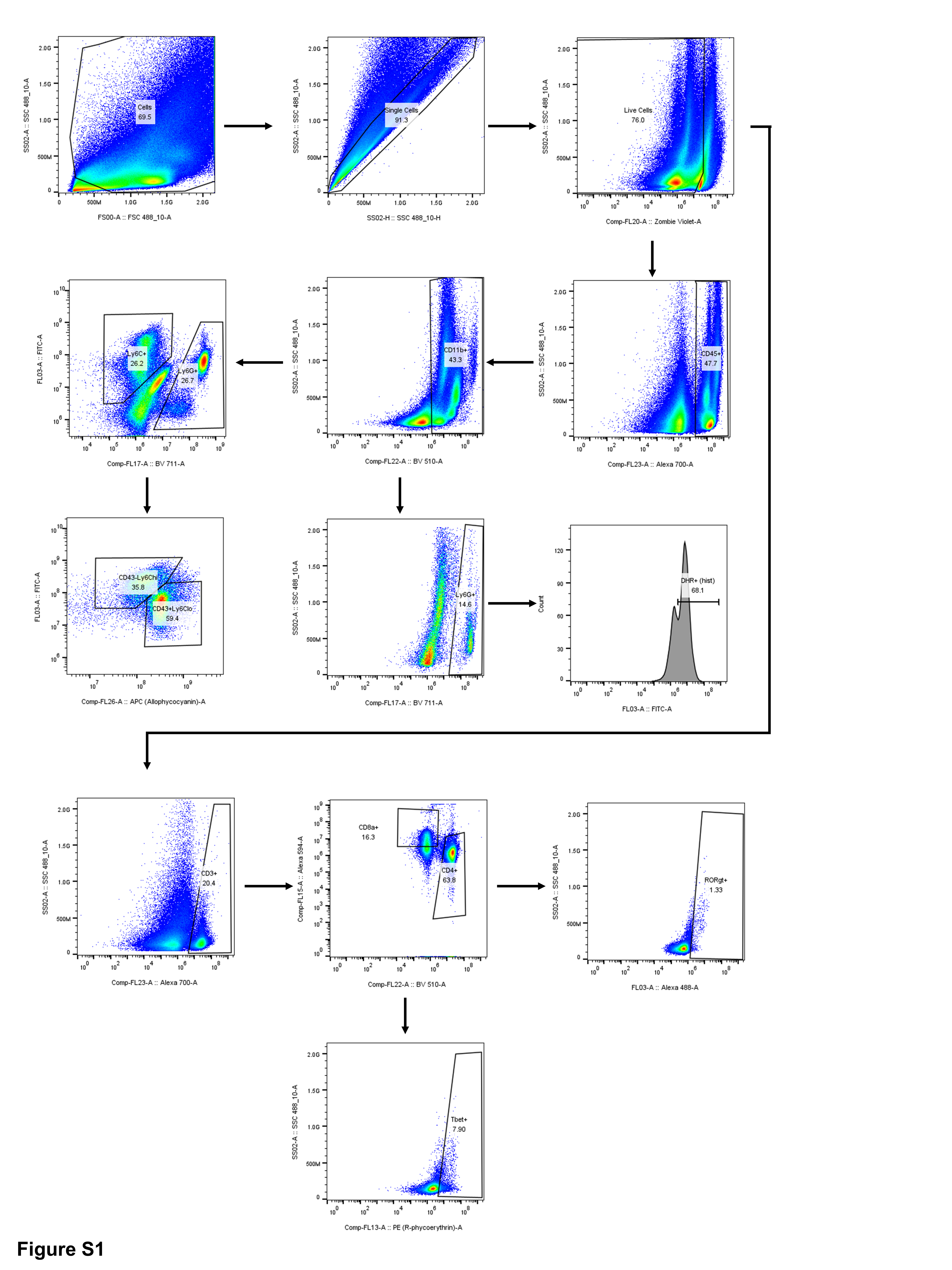

Supplement: Supplementary file 1 — Fig. S1. Gating scheme for flow cytometric analysis. [file MOL2-19-2860-s006.tif]

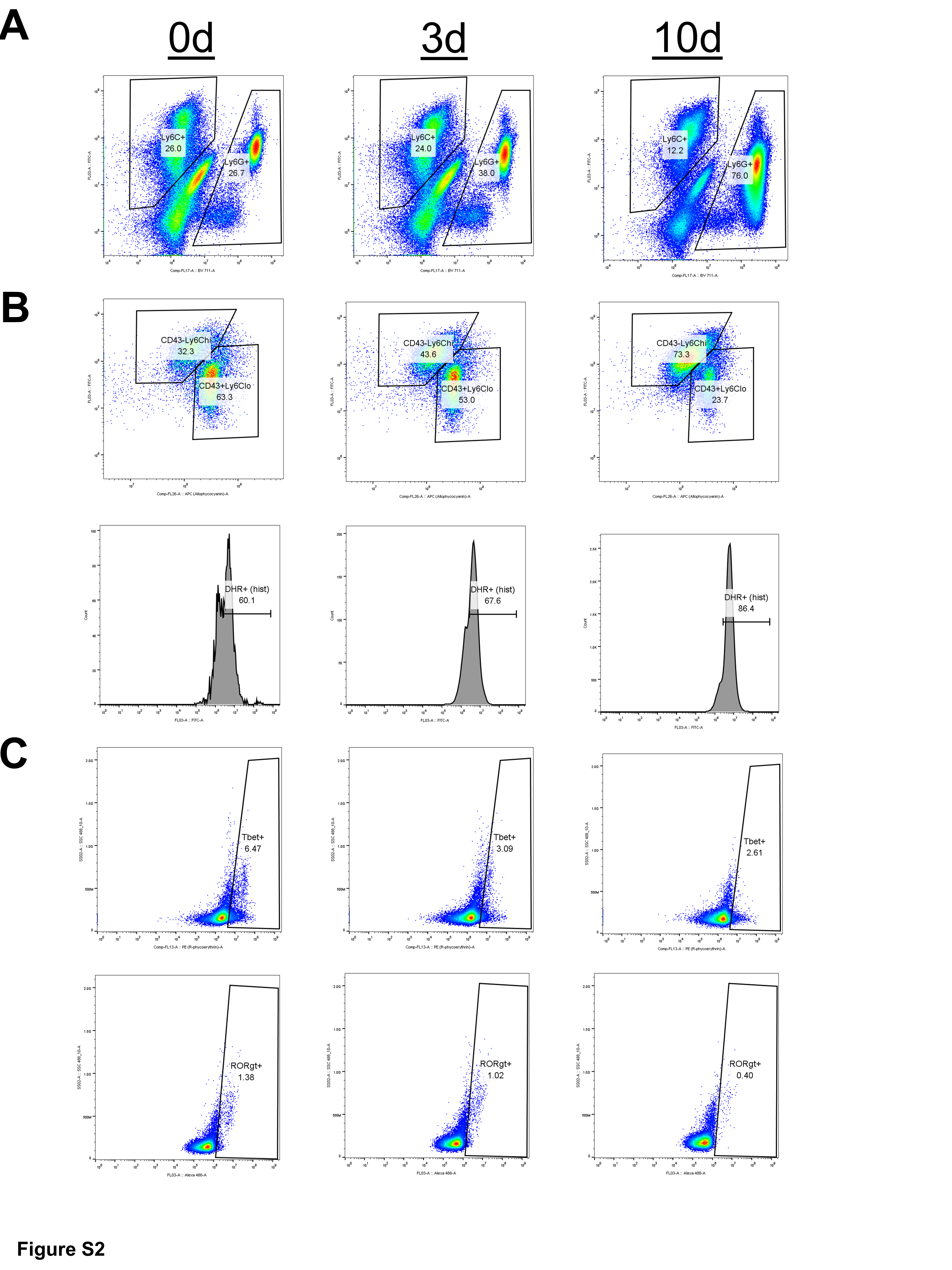

Supplement: Supplementary file 2 — Fig. S2. Representative images for flow cytometric analysis. (A) Representative images for Ly6C+/Ly6G+ populations among CD11b+ cells, corresponding to Fig. 1B. (B) Representative images for CD43+ populations among Ly6C+ cells, and reactive oxygen species (ROS) + populations among LyG6+ cells, corresponding to Fig. 1C. (C) Representative images for Tbet+ and RORγt+ populations among CD4+ cells, corresponding to Fig. 1E. [file MOL2-19-2860-s005.tif]

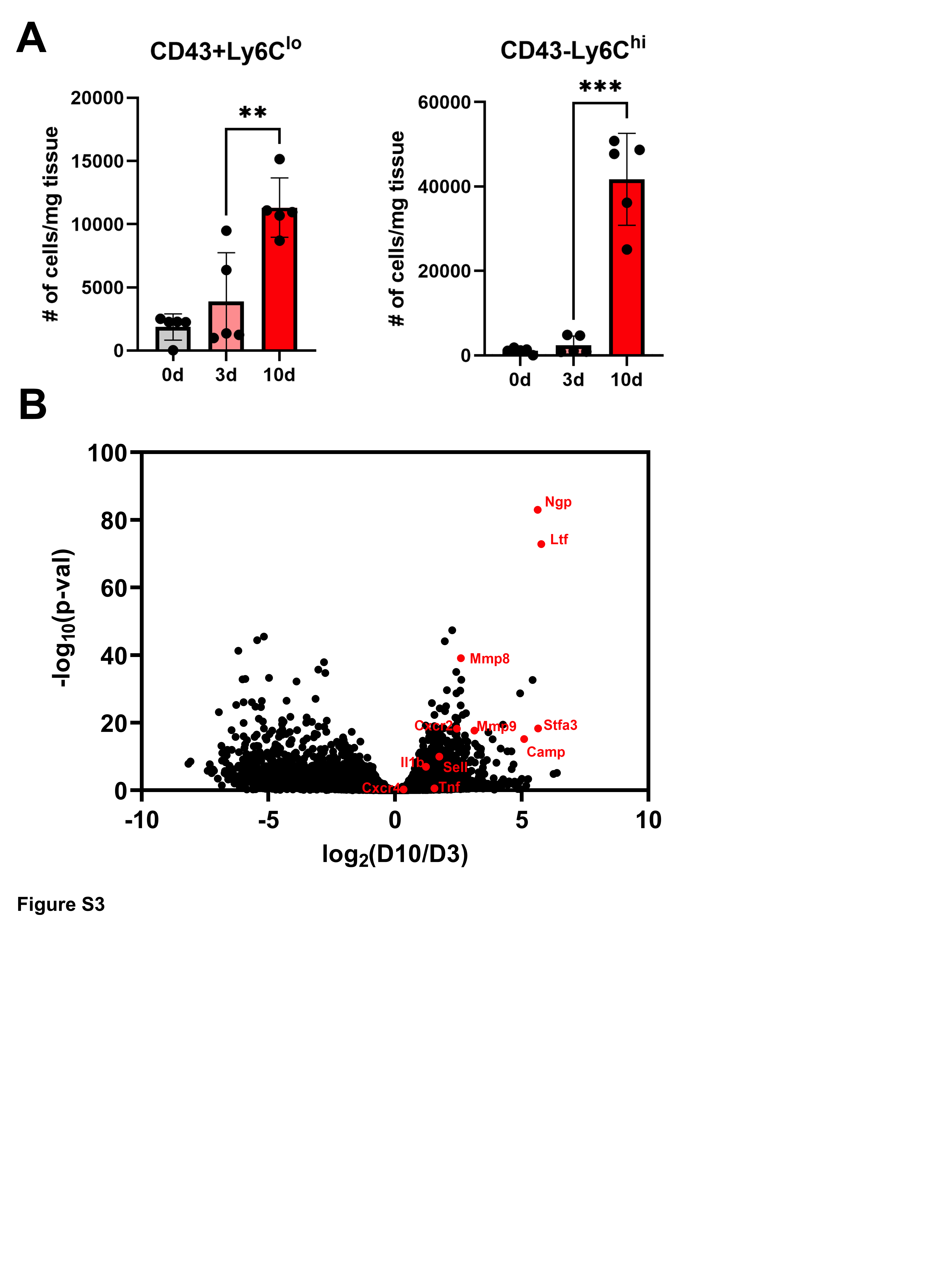

Supplement: Supplementary file 3 — Fig. S3. Absolute cell counts and gene expression of Gr1+ cells at 0 days (0d), 3 days (3d), and 10 days after inoculation (10d). (A) The number of classical monocytes per mg of lung tissue increases to higher levels than those of nonclassical monocytes. (B) Neutrophil‐associated genes are upregulated in Gr1+ cells from metastatic lungs compared to Gr1+ cells from premetastatic lungs. Two‐tailed unpaired t‐tests assuming unequal variance were performed for single comparisons between two conditions, **P ≤ 0.01, ***P ≤ 0.001. Bars indicate mean ± standard deviation with n = 5 biological replicates. [file MOL2-19-2860-s004.tif]

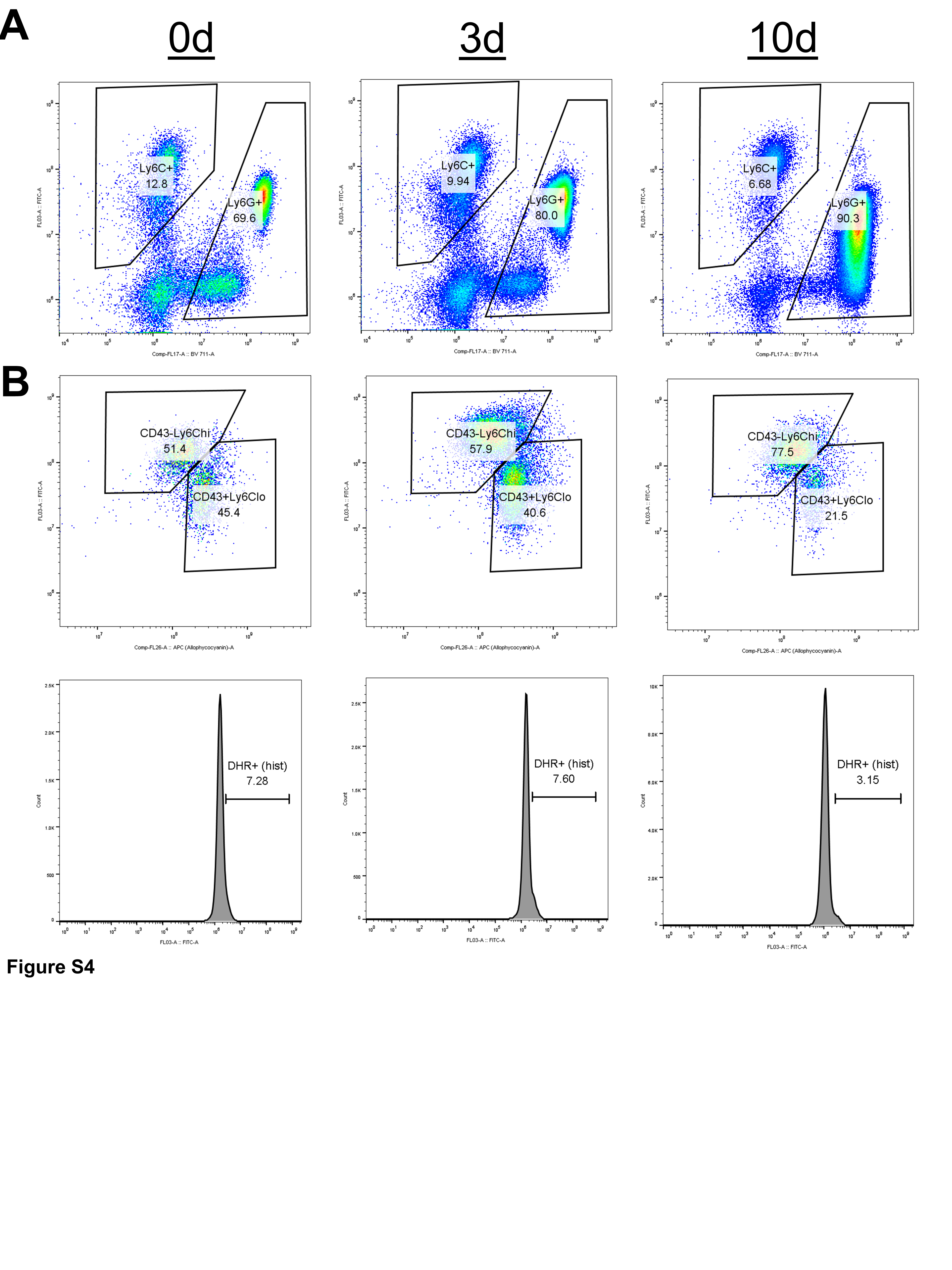

Supplement: Supplementary file 4 — Fig. S4. Representative images for flow cytometric analysis. (A) Representative images for Ly6C+/Ly6G+ populations among CD11b+ cells, corresponding to Fig. 2A. (B) Representative images for CD43+ populations among Ly6C+ cells, and reactive oxygen‐species expressing‐populations (dihydrorhodamine – DHR+) among LyG6+ cells, corresponding to Fig. 2C. [file MOL2-19-2860-s003.tif]

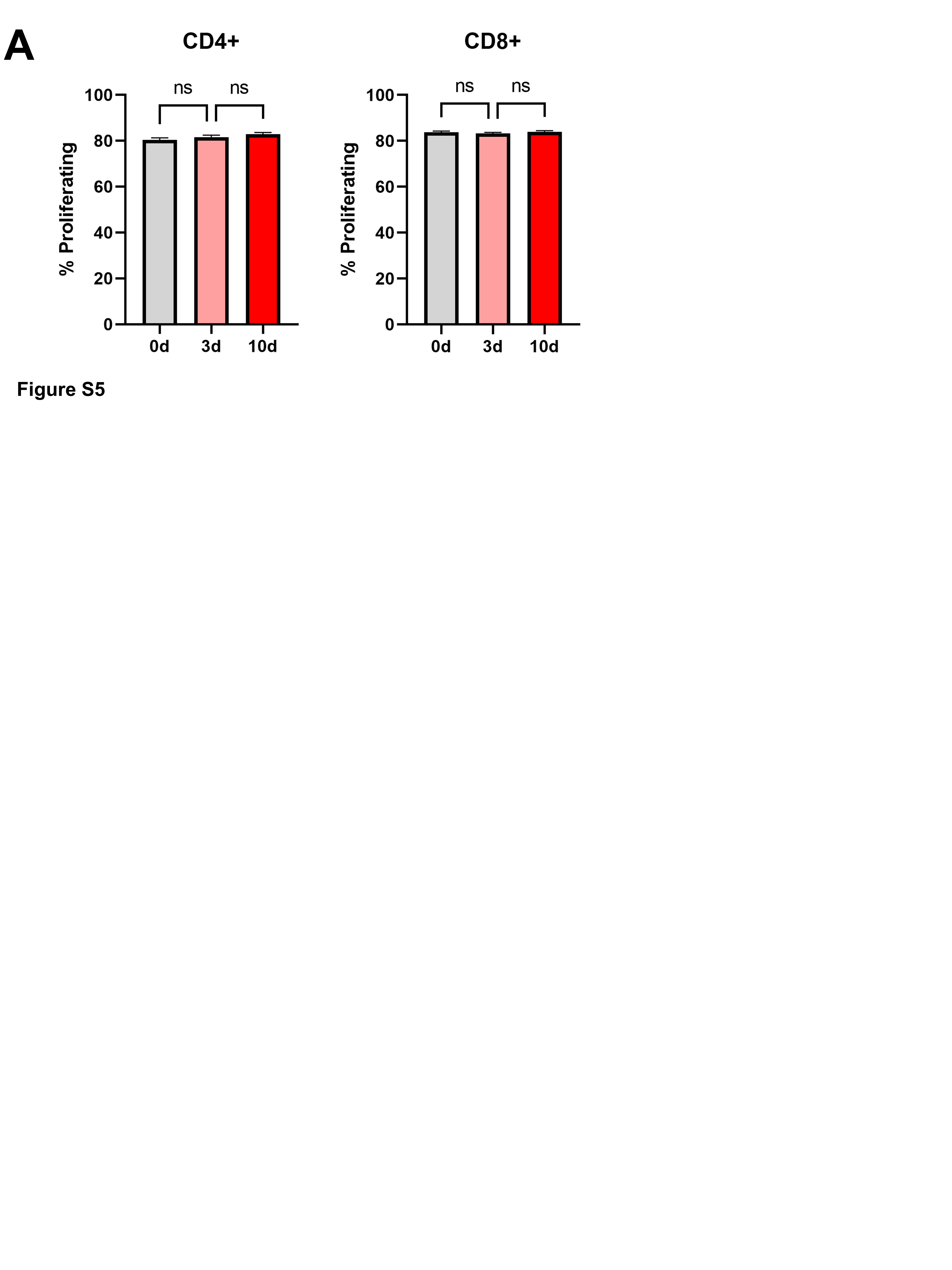

Supplement: Supplementary file 5 — Fig. S5. Gr1+ cell conditioned media from 0 days (0d), 3 days (3d), and 10 days after inoculation (10d) does not significantly influence T cell proliferation. Two‐tailed unpaired t‐tests assuming unequal variance were performed for single comparisons between two conditions, ns – no significance. Bars indicate mean ± standard deviation with n = 5 technical replicates. [file MOL2-19-2860-s010.tif]

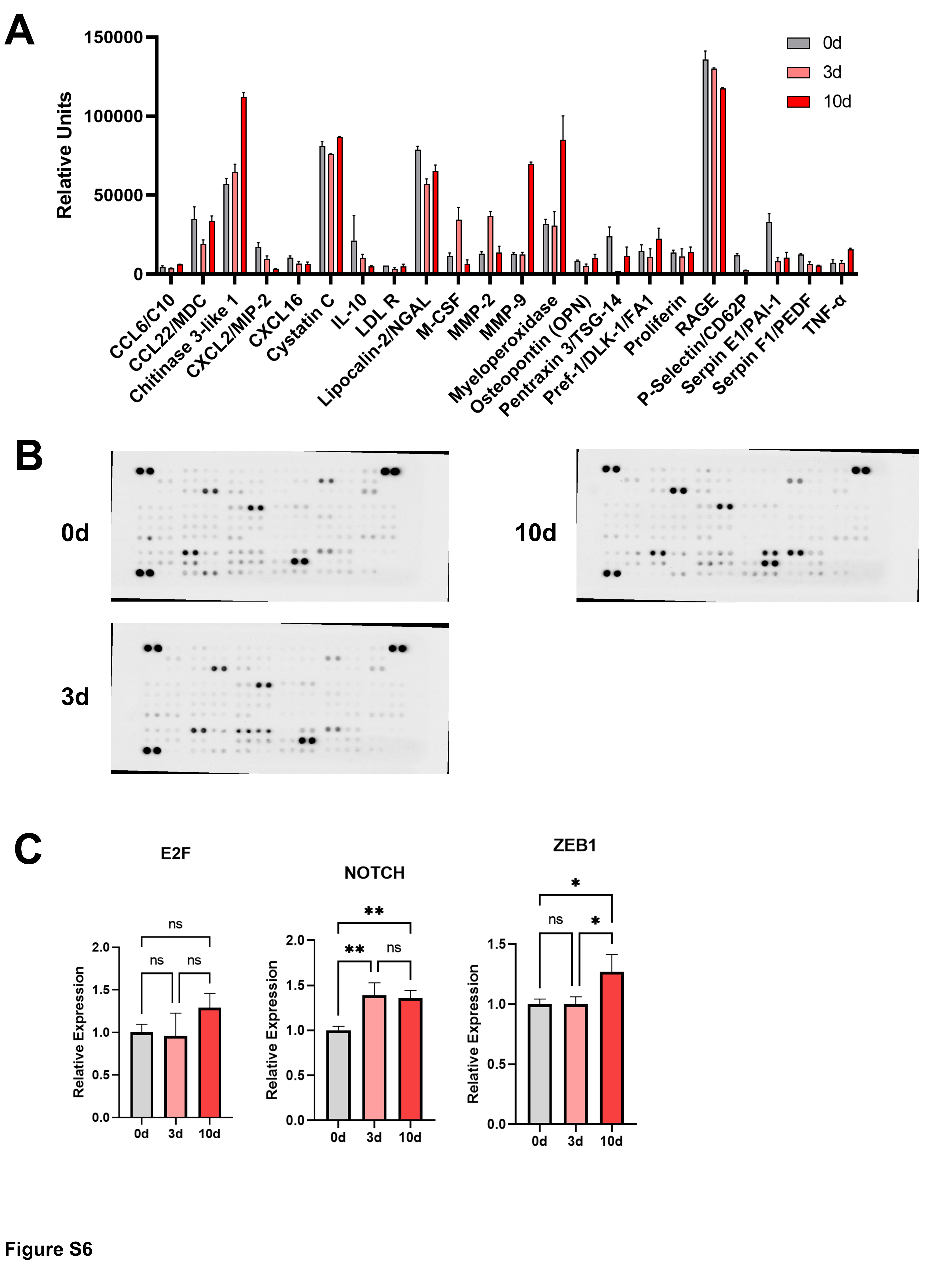

Supplement: Supplementary file 6 — Fig. S6. Secreted factors by Gr1+ cells modulate transcription factor activity in 4T1 cells. (A) Full panel of immunoassay proteins identified as highly secreted (> 5000 relative units). (B) Raw protein membrane array images. (C) Gr1+ cell conditioned media from 0 days (0d), 3 days (3d), and 10 days after inoculation (10d) altered activity of transcription factors that were not directly related to Chi3l1 signaling. Two‐tailed unpaired t‐tests assuming unequal variance were performed for single comparisons between two conditions, *P ≤ 0.05, **P ≤ 0.01. [file MOL2-19-2860-s011.tif]

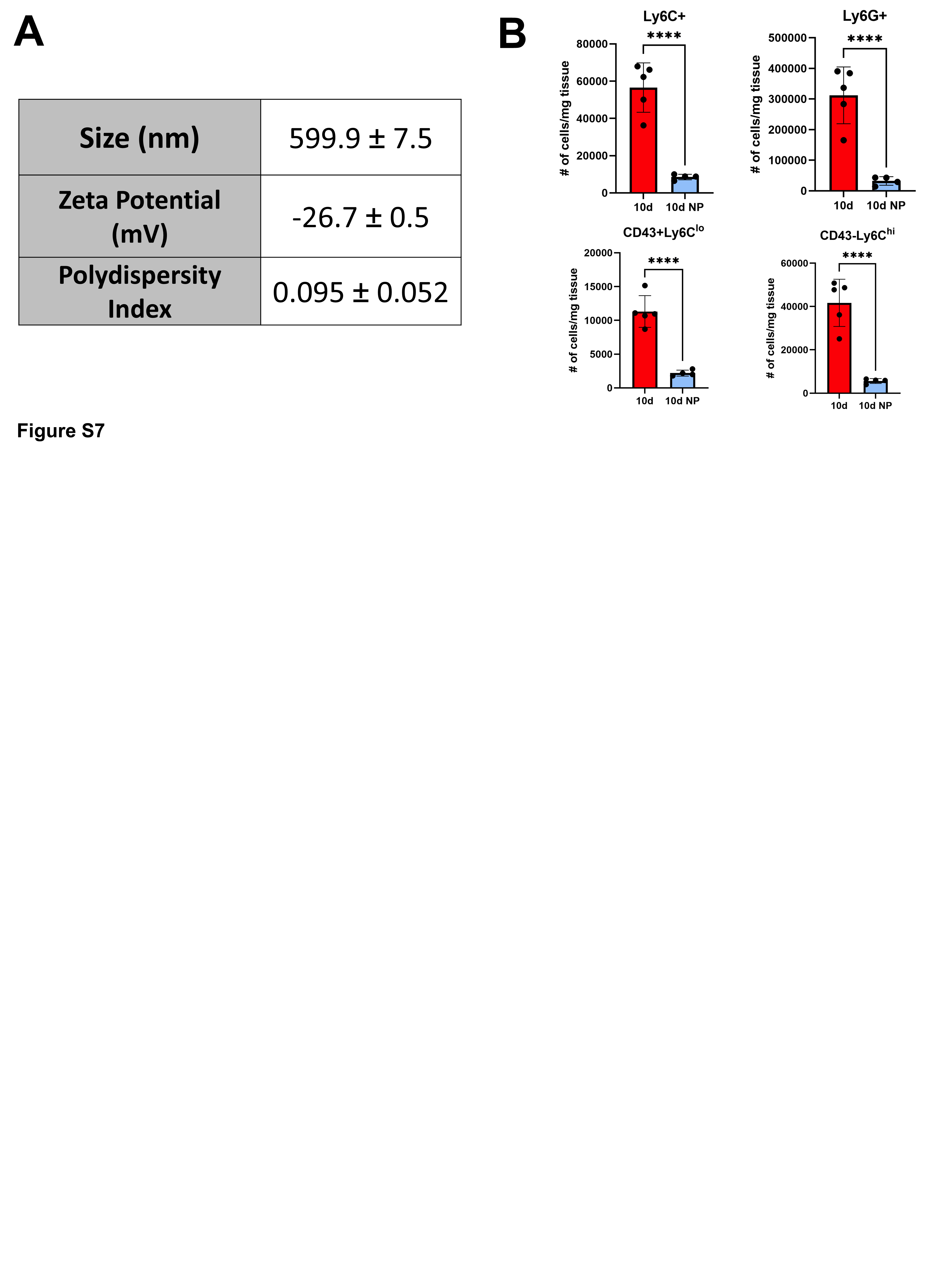

Supplement: Supplementary file 7 — Fig. S7. Nanoparticles (NP) reduce immune accumulation at the lung. (A) Nanoparticles are approximately 500 nm in diameter and have a negative surface charge. (B) Nanoparticles reduce the accumulation of myeloid cells, especially of neutrophils and classical monocytes compared to untreated mice 10 days after inoculation (10d). Two‐tailed unpaired t‐tests assuming unequal variance were performed for single comparisons between two conditions, ****P ≤ 0.0001. Bars indicate mean ± standard deviation with n = 5 biological replicates. [file MOL2-19-2860-s008.tif]

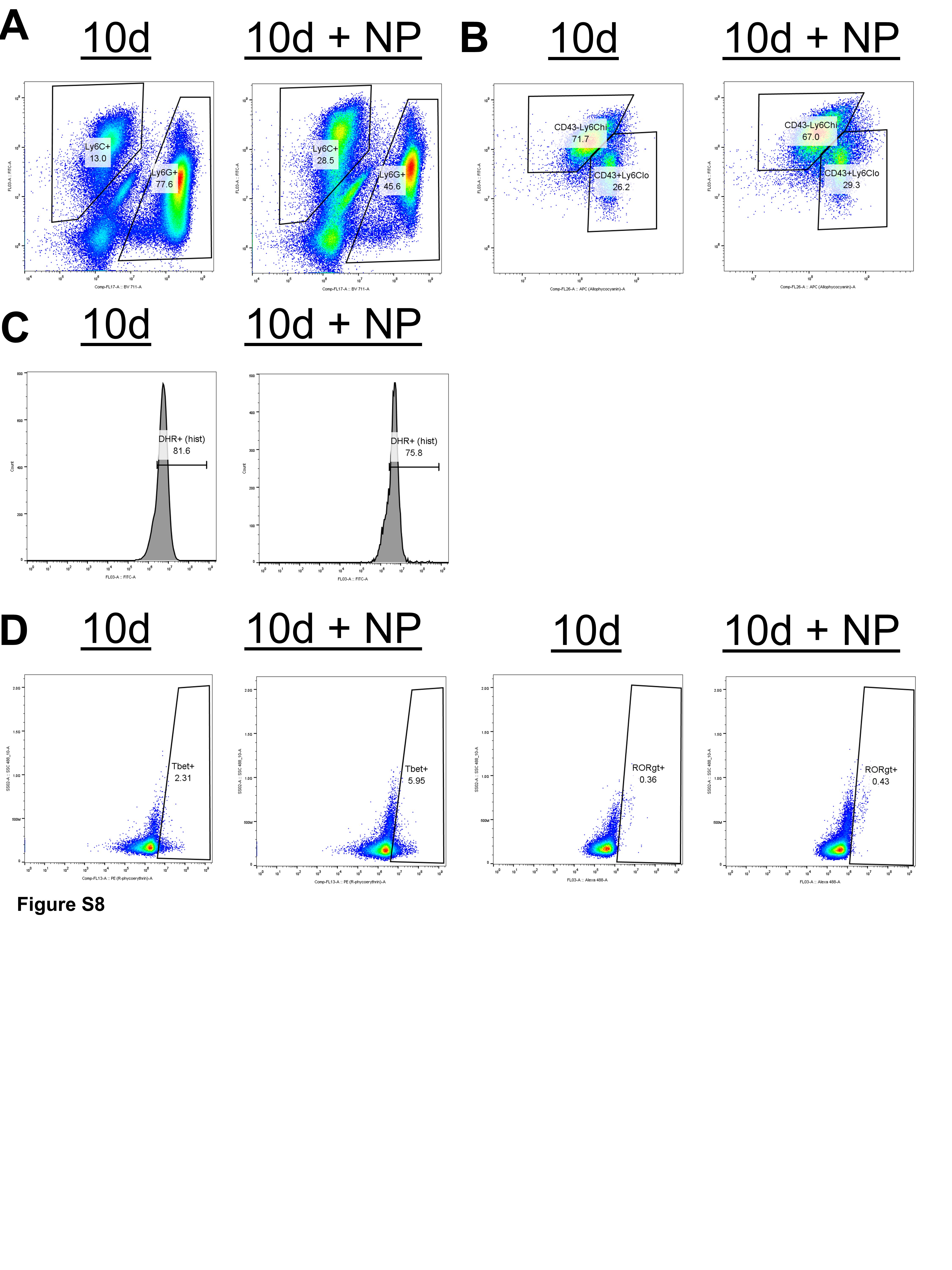

Supplement: Supplementary file 8 — Fig. S8. Representative images for flow cytometric analysis. (A) Representative images for Ly6C+/Ly6G+ populations among CD11b+ cells between untreated (10d) and nanoparticle‐treated mice (10d + NP), corresponding to Fig. 5B. (B) Representative images for CD43+ populations among Ly6C+ cells, corresponding to Fig. 5C. (C) Representative images for reactive oxygen species (ROS) + populations among Ly6G+ cells, corresponding to Fig. 5C. (D) Representative images for Tbet+ and RORγt+ populations among CD4+ cells, corresponding to Fig. S7B. [file MOL2-19-2860-s009.tif]

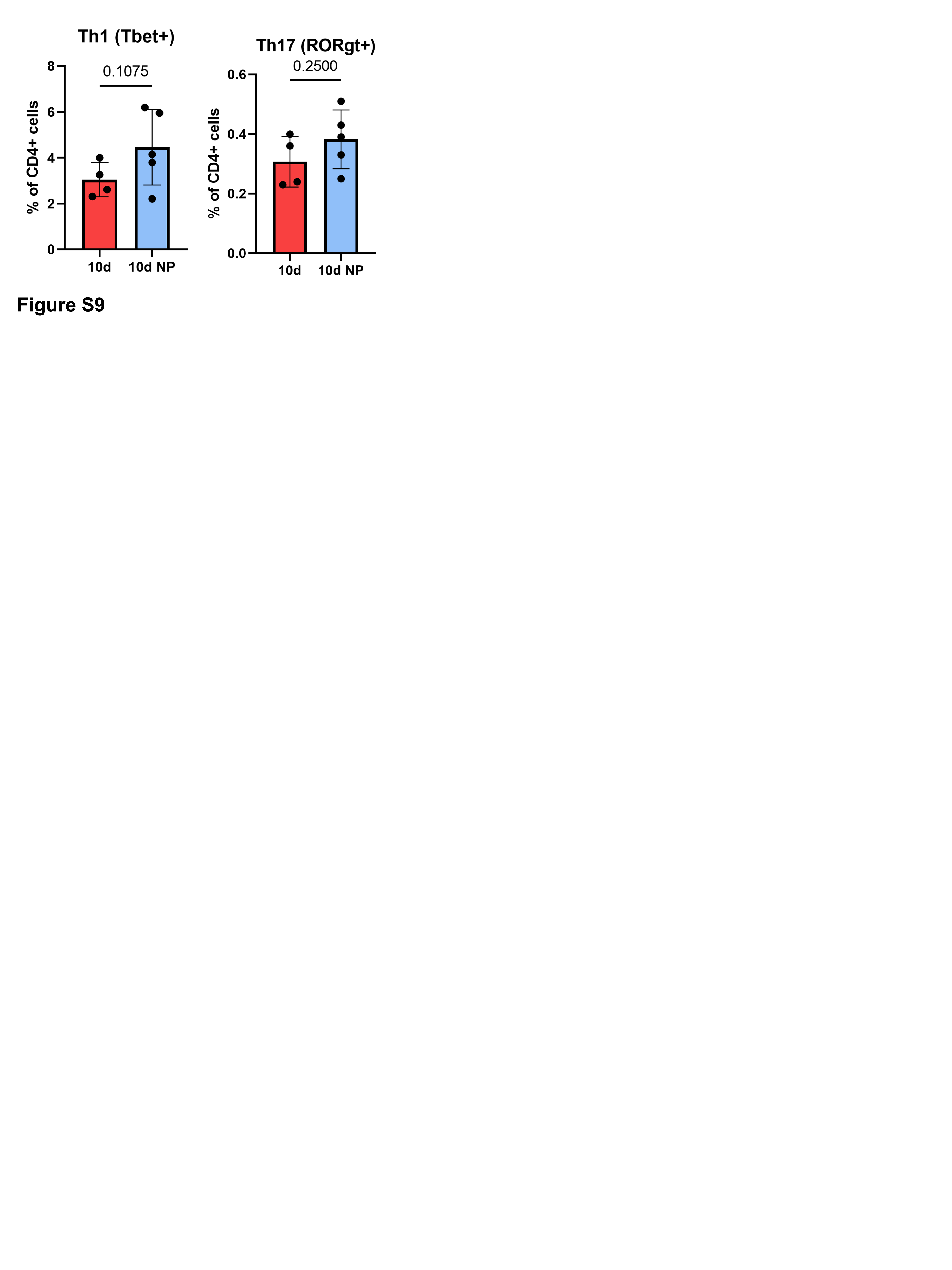

Supplement: Supplementary file 9 — Fig. S9. Nanoparticles (NP) drive a trend towards increased expression of Tbet and RORγt in CD4+ T cells in vivo in comparison to untreated mice 10 days after inoculation (10d). Two‐tailed unpaired t‐tests assuming unequal variance were performed for single comparisons between two conditions. Bars indicate mean ± standard deviation with n = 5 biological replicates. [file MOL2-19-2860-s001.tif]

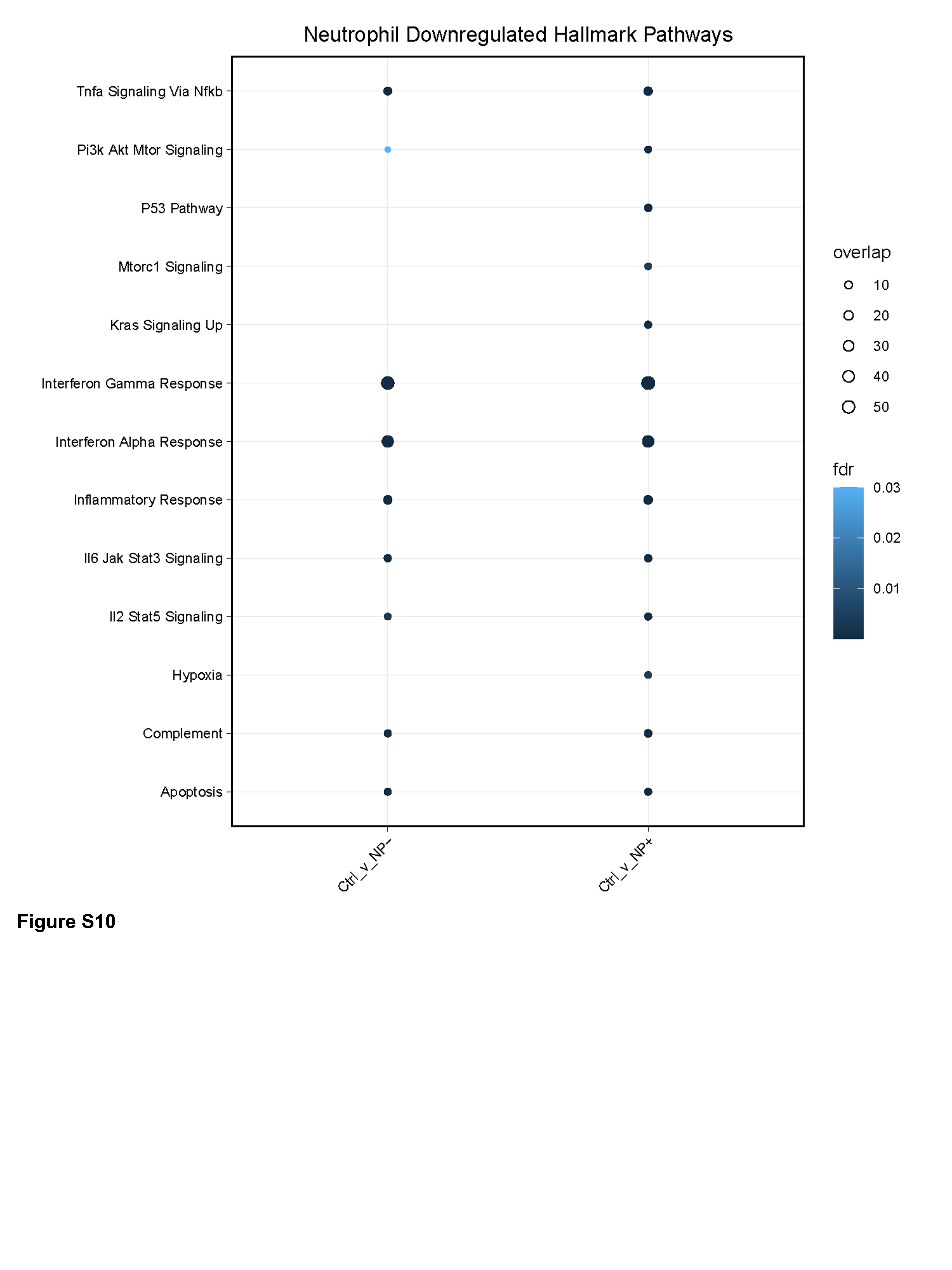

Supplement: Supplementary file 10 — Fig. S10. Single‐cell RNA sequencing demonstrates that nanoparticles broadly reprogram Gr1+ cells relative to cells in untreated mice. Nanoparticle treatment downregulates genes associated with JAK–STAT, interferon, and TGFβ signaling in both nanoparticle (NP)+ and NP− cells. The influence of nanoparticles on hypoxia, Kras, Mtorc1, and P53 is restricted to NP+ cells. [file MOL2-19-2860-s012.tif]

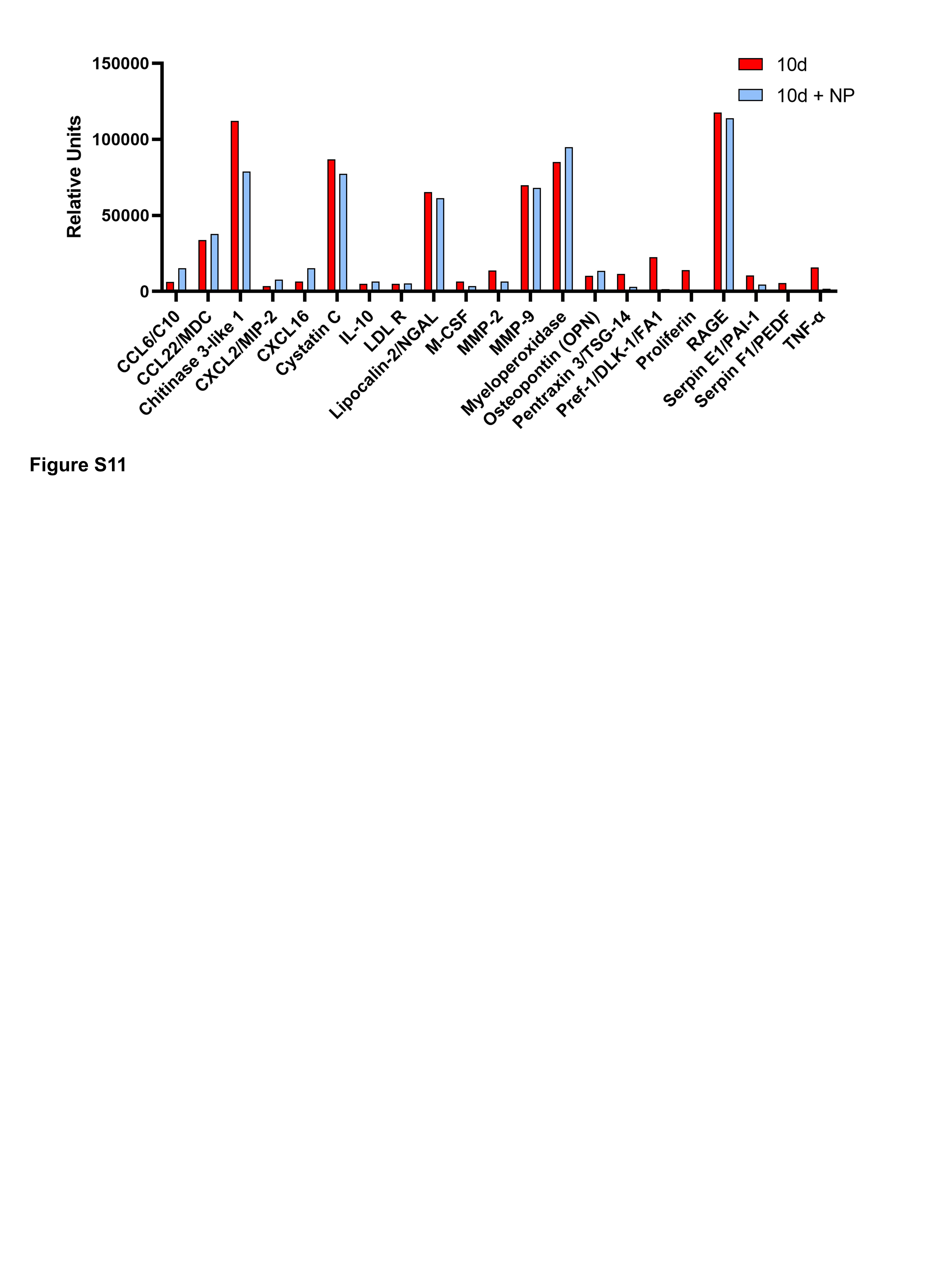

Supplement: Supplementary file 11 — Fig. S11. Full panel of immunoassay proteins identified as highly secreted in nanoparticle‐treated mice (> 5000 relative units). [file MOL2-19-2860-s002.tif]

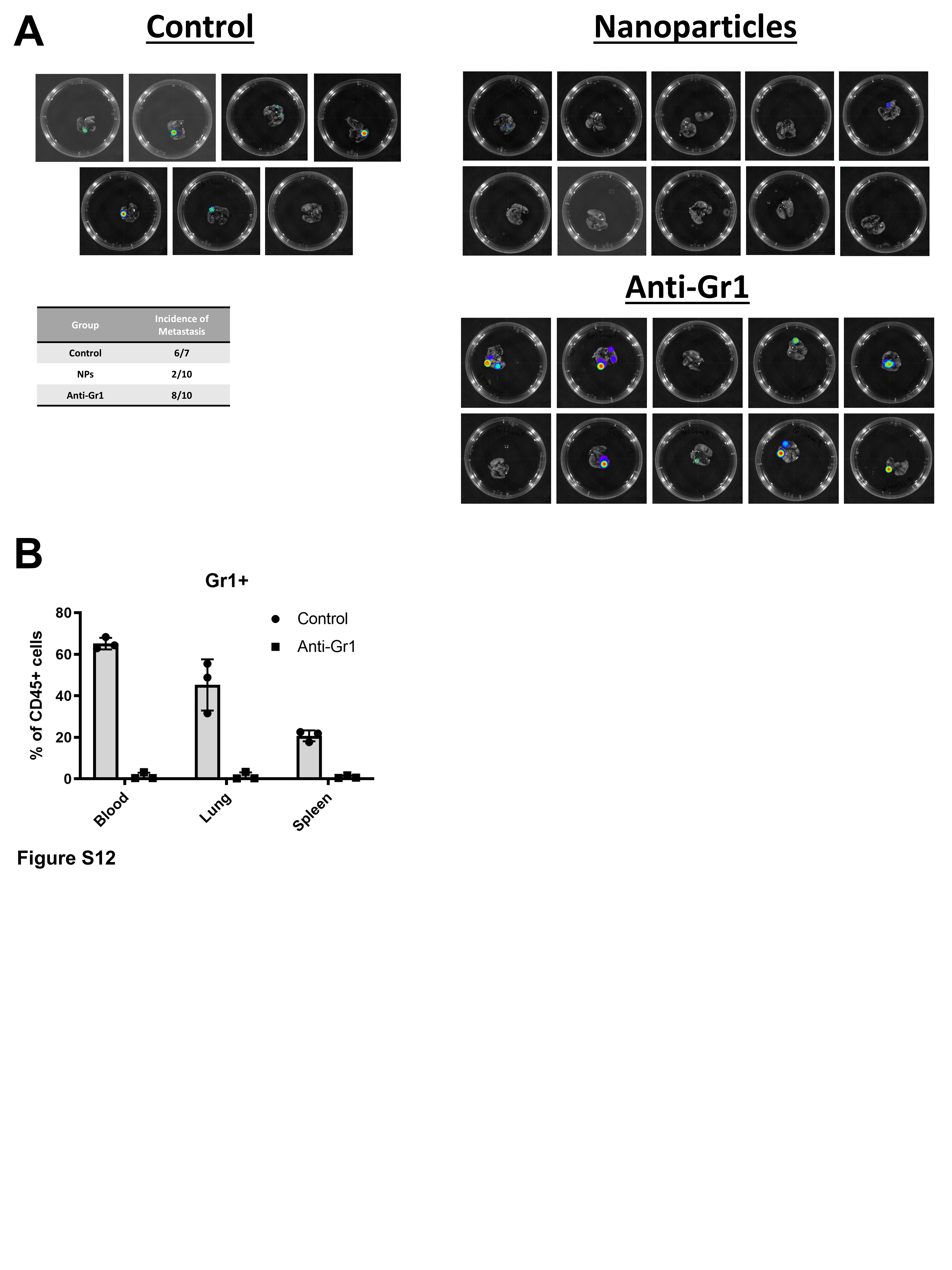

Supplement: Supplementary file 12 — Fig. S12. Nanoparticles (NP), but not anti‐Gr1, inhibit lung metastasis. (A) Nanoparticles, but not anti‐Gr1, reduce the incidence of lung metastasis. (B) Anti‐Gr1 treatment reduces Gr1 cells to levels undetectable by flow cytometry in the lung, blood, and spleen. Two‐tailed unpaired t‐tests assuming unequal variance were performed for single comparisons between two conditions. Bars indicate mean ± standard deviation with n = 3 biological replicates. [file MOL2-19-2860-s007.tif]
